# Experimental Research on Online Monitoring of Crack Evolution Process of π-Type Beams Based on Ultra-Weak FBG Array Sensing Technology

**DOI:** 10.3390/s26154779

**Published:** 2026-07-27

**Authors:** Qiuming Nan, Yichan Zhang, Juncheng Zeng, Sheng Li, Lina Yue, Yan Yang, Min Zhou, Qi Hu

**Affiliations:** 1 National Engineering Research Center of Fiber Optic Sensing Technology and Networks, Wuhan University of Technology, Wuhan 430070, China; nqm0723@whut.edu.cn (Q.N.); lisheng@whut.edu.cn (S.L.); linayue@whut.edu.cn (L.Y.); 2 School of Mechanical and Electronic Engineering, Wuhan University of Technology, Wuhan 430070, China; zychan@whut.edu.cn; 3 Fujian Provincial Expressway Technology Innovation Research Institute Co., Ltd., Fuzhou 350001, China; ulzeng@126.com; 4 Fengli Optoelectronics Technology Co., Ltd., Wuhan 430070, China; zhoumin@wutos.com; 5 School of Information Engineering, Wuhan University of Technology, Wuhan 430070, China; hqiwyyx@163.com

**Keywords:** π-shaped beam, grating array, crack evolution, online monitoring

## Abstract

Traditional crack monitoring methods, relying on discrete point sensors, cannot capture the full spatiotemporal evolution of cracks. To address this limitation, this paper presents a distributed online monitoring approach using ultra-weak Fiber Bragg Grating (UWFBG) array sensing technology. A 16 m full-scale π-beam was instrumented with a grating array strain sensing system and tested under progressive mid-span loading until failure. The array successfully detected crack initiation at 848.7 kN (0.9P1) and tracked the transformation from L-shaped to U-shaped cracks, yielding a final crack count of 90 with a maximum width of 1.21 mm and length of 246.5 cm at 1791.7 kN. The strain–load curves exhibited a clear linear-to-nonlinear transition and continuous slope increase, closely matching manual observations. Quantitative correlation analysis further established a strong linear relationship between UWFBG peak strains and manually measured crack widths, with the fitting equation ε=7918·w−110 and a coefficient of determination R^2^ = 0.971, providing a specimen-specific basis for strain-based crack severity estimation that requires in-situ calibration before field application. The UWFBG array maintained stable signal acquisition throughout the entire loading process, offering superior data continuity and measurement range compared to resistive strain gauges, which suffered progressive data loss after cracking. The results demonstrate that the proposed method can provide real-time, full-field strain mapping and quantitative crack evolution monitoring, offering a powerful tool for bridge health assessment.

## 1. Introduction

Highway bridges constitute core transportation infrastructure, whose structural safety and service performance directly determine the stable operation of road networks [1]. The Changshengkou Bridge, as a key node of the expansion and renovation project of the Shanghai–Wuhan Expressway from Wuhan to Yichang, extensively adopts pre-tensioned prestressed concrete π-shaped beams for its superstructure. This type of component has advantages such as moderate section stiffness, convenient construction, and good economy, and is widely used in simply supported beam bridges of highways [2]. At the junction of the web plate and the bottom plate of the π-shaped beam, as well as in the tension zone at the mid-span, there is a stress concentration phenomenon. Under the action of construction loads, vehicle loads, and long-term environmental factors, initial cracks are likely to occur and gradually expand, eventually forming a through-crack, which reduces the beam’s flexural bearing capacity and structural durability. Therefore, realizing online monitoring of the entire process of π-shaped beam crack evolution has significant engineering significance for bridge safety warning [3].

At present, the primary techniques employed for structural crack monitoring can be classified into four categories: mechanical crack measurement, resistance strain gauges, ultrasonic testing, and conventional point-type fiber optic sensing. However, each of these methods suffers from intrinsic limitations that render them insufficient for monitoring the full life-cycle crack evolution process in prestressed π-shaped beams.

1. Mechanical crack measurement. This approach relies on handheld devices such as mechanical crack gauges, optical crack comparators, or digital microscopes to manually record crack width and length at discrete time intervals. The method is inherently labor-intensive and cannot provide real-time or automated data acquisition. It is limited to surface-visible cracks and cannot detect internal cracking or subsurface propagation. Most critically, during destructive loading tests, personnel are prohibited from approaching the danger zone, making it impossible to capture crack development at high load levels where the most significant structural damage occurs. The data are temporally sparse and cannot reflect the continuous evolution path of cracks from initiation to failure.
2. Resistance strain gauges. Strain gauges are widely used due to their low cost and simple installation. Nevertheless, they are fundamentally point sensors, each measuring strain only over a small localized area (typically 5–20 mm in gauge length). To monitor a beam, a prohibitively large number of gauges would be required to achieve reasonable spatial coverage, leading to extensive wiring complexity and increased cost. More importantly, when a crack propagates directly through or adjacent to a bonded strain gauge, the gauge frequently debonds, fractures, or exceeds its measurement range, resulting in irreversible data loss. This failure predominantly occurs in the plastic deformation stage when crack information is most valuable. As a result, strain gauges cannot provide the continuous, full-field strain profiles necessary for tracking crack-induced stress redistribution across the entire beam.
3. Ultrasonic testing. Ultrasonic pulse velocity (UPV) and phased-array techniques can detect internal flaws and estimate crack depth. However, these methods are primarily suited for periodic, localized inspections rather than continuous, global monitoring. They require skilled operators, specialized coupling agents, and stable environmental conditions. For a prestressed π-beam with a complex cross-sectional geometry (including thin webs, wide bottom slabs, and internal prestressing tendons), acoustic wave propagation paths are complicated, and signals are affected by concrete heterogeneity, aggregate scattering, and reinforcement reflections, leading to limited spatial resolution and ambiguous interpretation of crack dimensions. Furthermore, ultrasonic testing cannot be performed simultaneously with structural loading in a continuous manner, making it unsuitable for real-time crack tracking during destructive tests.
4. Conventional point-type fiber optic sensing (discrete FBGs). Discrete FBGs offer advantages over electrical sensors, including immunity to electromagnetic interference, corrosion resistance, and multiplexing capability. Despite these benefits, each discrete FBG measures strain only at a single point. The spatial resolution is entirely determined by the number and spacing of the gratings deployed. Cracks occurring between adjacent FBGs are likely to be missed or inaccurately characterized. While wavelength-division multiplexing (WDM) allows several gratings to share a single fiber, the total number of sensors per channel is typically limited to fewer than 20 for static strain measurements, which is far from sufficient to achieve distributed coverage over the full length of a 16 m beam. Moreover, discrete FBGs cannot reconstruct a continuous strain field profile; they provide only isolated strain values that fail to capture the spatial gradient and stress redistribution characteristics associated with crack propagation.

Collectively, these traditional methods share a fundamental deficiency: they cannot provide long-distance, spatially continuous, real-time, and full-time-domain strain field data—the very type of information required for comprehensively characterizing the dynamic evolution process of cracks in prestressed π-shaped beams from initiation through propagation to ultimate failure [4]. This gap motivates the adoption of UWFBG array sensing technology, which combines the advantages of distributed measurement, high sensitivity, electromagnetic immunity, and adaptability to large-deformation conditions [5]. It can realize continuous global strain collection via a single optical cable and accurately capture strain mutations and stress redistribution induced by cracks, thus offering technical support for online monitoring of crack evolution in π-shaped beams [6].

In recent years, distributed optical fiber sensing (DOFS) technologies have attracted considerable international research attention as a promising alternative for crack monitoring in concrete structures. Comprehensive reviews by Bado and Casas [7] and by the TU Delft group [8] systematically surveyed the application of Rayleigh scattering-based (e.g., optical frequency domain reflectometry, OFDR), Brillouin scattering-based, and Raman scattering-based techniques for civil structural health monitoring, confirming that DOFS can achieve strain measurement with spatial resolutions as fine as 0.63 mm and are capable of detecting, locating, and quantifying cracks. Laboratory studies on reinforced concrete beams have demonstrated that DOFS tools can identify crack-induced localized strain peaks and estimate crack widths through strain integration, with results validated against digital image correlation (DIC) and electrical strain gauges [9,10]. Comparative research by Bednarski et al. [11] further revealed that monolithic composite DFOS sensors outperform conventional layered sensing cables in terms of crack shape coefficient and quantitative width estimation, and proposed practical guidelines for sensor selection in field applications.

Parallel to the advancement of general DOFS methods, UWFBG array technology has emerged as a particularly attractive solution for large-scale, high-density quasi-distributed strain sensing. By adopting a low-reflectivity grating design (reflectivity typically <0.1%), thousands of FBGs can be multiplexed on a single optical fiber using time-division multiplexing (TDM) combined with WDM, achieving a sensing network with hundreds to thousands of measurement points over kilometer-scale distances [12,13]. Recent experimental investigations have validated the effectiveness of UWFBG arrays for strain distribution measurement in prestressed concrete beams [6], for bridge strain monitoring under operational loads [9], and for highway subgrade structural health assessment [11]. Pan et al. [10] further proposed a long-gauge ultra-weak FBG strain sensor that is insensitive to local structural defects, enhancing the reliability of crack-induced strain measurements. The core advantage of the UWFBG array—its ability to provide continuous, quasi-distributed strain profiles along the entire instrumented length while maintaining the high sensitivity and multiplexing density of FBG technology—makes it uniquely suited for monitoring the full crack evolution process in full-scale prestressed concrete bridge components.

In summary, although FBG-based sensors and distributed optical fiber sensing have been extensively applied in structural health monitoring, the existing studies rarely address full-scale pretensioned prestressed π-shaped beams, nor do they achieve distributed online tracking of the entire cracking process from initiation to failure. This work fills that gap by making the following distinct contributions:

1. It is the first to deploy a UWFBG array on a 16 m full-scale prestressed concrete π-beam, enabling continuous, distributed strain monitoring over the whole beam length and throughout the whole loading history;
2. It reveals that characteristic spatiotemporal strain patterns—specifically a triangular-shaped strain distribution and a linear-to-nonlinear transition—can reliably indicate crack initiation, propagation, and ultimate failure;
3. It provides a systematic comparison with conventional resistance strain gauges and theoretical predictions, demonstrating that the UWFBG array offers broader measurement range, higher reliability, and better data continuity under large deformations;
4. It establishes a quantitative relationship between FBG strain peaks and manually measured crack widths. These novelties collectively demonstrate the applicability and robustness of ultra-weak UWFBG arrays for crack monitoring of prestressed π-beams, thereby offering a practical technical reference for health monitoring of similar prefabricated bridge girders.

## 2. Theoretical Foundation

### 2.1. Grating Measurement Principle

The sensing core of the fiber Bragg grating (FBG) is a periodic refractive index modulation structure formed within the fiber core. When a broadband light source is incident onto the grating area, the specific wavelengths that meet the Bragg condition will be reflected, while the remaining wavelengths will be transmitted. The central reflected wavelength satisfies [14]:

(1) $$\lambda_{B}=2n_{eff}\Lambda$$

where $\lambda_{B}$ represents the central wavelength of the optical fiber grating; $n_{eff}$ is the effective refractive index of the optical fiber core; Λ is the grating period.

When the structure of the optical fiber undergoes axial strain or temperature changes, the grating period and the effective refractive index change, which in turn causes the central wavelength to shift. Under the condition of constant temperature, the wavelength shift is linearly related to the strain [15].

The UWFBG array adopts a low-reflection grating design, enabling the multiplexing of thousands of measurement points on a single optical fiber, achieving high-density distributed strain measurement. Compared with traditional FBG point-type sensors, the UWFBG array has the characteristics of a wide monitoring range, high spatial resolution, and suitability for long-distance structural monitoring [16]. It can precisely capture the strain gradient mutations around cracks, providing a data basis for crack location and width identification [17].

**Figure 1:**
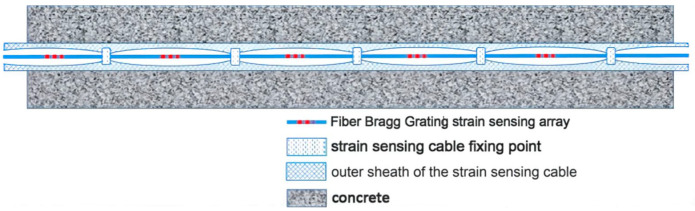
Schematic diagram of the grating array strain sensing optical cable structure.

Figure 1 shows the schematic diagram of the grating array strain sensing optical cable structure. As can be seen from the figure, the sensing optical cable is composed of several consecutive sensing units, and each sensing unit is equivalent to a strain sensor with a 1 m measurement range, measuring the average strain within the measurement range. The strain measured by each sensing unit forms a continuous strain field [18]. Compared with traditional point-type strain gauges, the grating array sensing cable has the advantages of simple layout, reliable positioning, and stable operation throughout the entire length, which can make up for the deficiency of insufficient spatial coverage of traditional point-type monitoring [19].

When a π-shaped beam cracks, the crack tip experiences stress concentration, causing the strain around the concrete to increase sharply. The grating array can identify the location of crack initiation based on the characteristic of wavelength mutation. As the crack extends, the strain gradient changes along the extension direction of the crack, and the grating array can track the crack expansion path and width changes in real time, establishing a quantitative correlation between the wavelength offset and the crack parameters [20].

It should be noted that the wavelength shift of an FBG is theoretically affected by both strain and temperature. However, in this experiment, no additional temperature-compensating reference gratings or active temperature correction algorithms were employed. This decision was based on the following considerations: throughout the entire stepwise loading process, the ambient temperature varied very slowly and the total fluctuation was strictly controlled within 2 °C. For an FBG with a central wavelength of 1550 nm, a 2 °C temperature change corresponds to a strain equivalent of only approximately 20–30 με. In contrast, the strain responses induced by the applied loads in this test ranged from several hundred micro-strains in the elastic stage to several thousand micro-strains at failure, with the mechanical strain being absolutely dominant. The temperature-induced wavelength drift corresponds to an apparent strain of approximately 20–30 με, which is larger than the nominal resolution of the interrogator (≈0.83 με) but still constitutes less than 5% of the minimum elastic strain response measured in this test. Throughout the entire loading sequence, the mechanical strain signal dominated by at least an order of magnitude. Therefore, under the specific stable temperature conditions of this experiment, the temperature effect does not distort the crack evolution analysis and was safely neglected without active compensation, and it does not substantially affect the analysis of crack evolution patterns or the overall strain–load curve trends. Therefore, under the specific stable temperature conditions of this experiment, the temperature effect can be safely neglected without the need for additional compensation.

In the test, the raw data recorded by the interrogator are the real-time central wavelength shifts ${∆}_{\lambda_{\beta }}$ (unit: pm) of each grating. To obtain the structural strain, these shifts must be converted into strain values ${∆}_{\varepsilon }$. In this experiment, an offline manual conversion was adopted, using the following formula:

(2) $${∆}_{\varepsilon }=\frac{{∆}_{\lambda_{\beta }}}{K_{\varepsilon }}$$

where $K_{\varepsilon }$ is the strain sensitivity coefficient, representing the wavelength shift induced by unit strain. The $K_{\varepsilon }$ value for this batch of UWFBG array cables was individually calibrated by the manufacturer before delivery, with a certified reference value of 1.2 pm/με (at a center wavelength of 1550 nm). In practice, the measured wavelength shift ${∆}_{\lambda_{\beta }}$ of each grating under each loading level was substituted into Equation (2) to obtain the corresponding strain value. This conversion process was performed offline after the test using data processing software in a batch mode, ensuring accuracy and traceability of the data processing.

### 2.2. Strain Calculation Model for π-Type Beams Under Elastic Loading Conditions

This test involves an elastic loading of concentrated load at the mid-span of a 16-m simply supported π-shaped beam. Based on the force theory of simply supported beams in material mechanics and the assumption of a flat cross-section, a strain calculation model for the elastic stage is derived.

Calculation of mid-span bending moment: When a concentrated load P acts on the mid-span of a simply supported beam, the bending moment M at the mid-span section is:

(3) $$M=\frac{PL}{4}$$

where L represents the calculated span of the beam; P represents the concentrated load applied at the mid-span.

Calculation of cross-sectional flexural rigidity: For the tension zone of the π-shaped beam, the distance from the edge to the centroidal axis is y, the moment of inertia of the cross-section is I, and the elastic modulus of the C50 concrete in the elastic stage is Ec. Then, the cross-sectional flexural rigidity B is:

(4) $$B=E_{c}I$$

Elastic strain theory formula: Based on the assumption of a flat cross-section and the strain–stress relationship in materials mechanics, the strain ε of the tension zone of concrete in the elastic stage satisfies:

(5) $$ℇ=\frac{My}{E_{c}I}$$

Substituting Equation (3) into Equation (5), the final strain–load calculation model for the tension zone at the mid-span of the π-shaped beam under elastic loading is obtained:

(6) $$ℇ=\frac{PLy}{{4E}_{c}I}$$

From Equation (6), it can be seen that the strain ε of the elastic stage of concrete has a strict linear relationship with the load P. The strain increases uniformly with the load, and the slope remains constant. There are no inflection points or sudden changes. This is the core mechanical characteristic of the beam when it has not cracked [21]; once the concrete cracks, the stress distribution in the section changes, the stiffness decreases, and the strain–load relationship will deviate from linearity and present a nonlinear rapid growth characteristic [22].

### 2.3. Force Division and Characteristics of π-Type Beams Throughout the Entire Process

Under the concentrated step loading at the mid-span of prestressed π-shaped beams, the entire process from elastic deformation to complete failure can be divided into the elastic stage, the crack initiation stage, the crack propagation stage, the plastic development stage, and the complete failure stage [23,24]. The corresponding force characteristics and strain–load curve variation patterns of each stage are shown in Table 1.

## 3. Structural Calculation and Test Design

### 3.1. Test Overview

**Figure 2:**
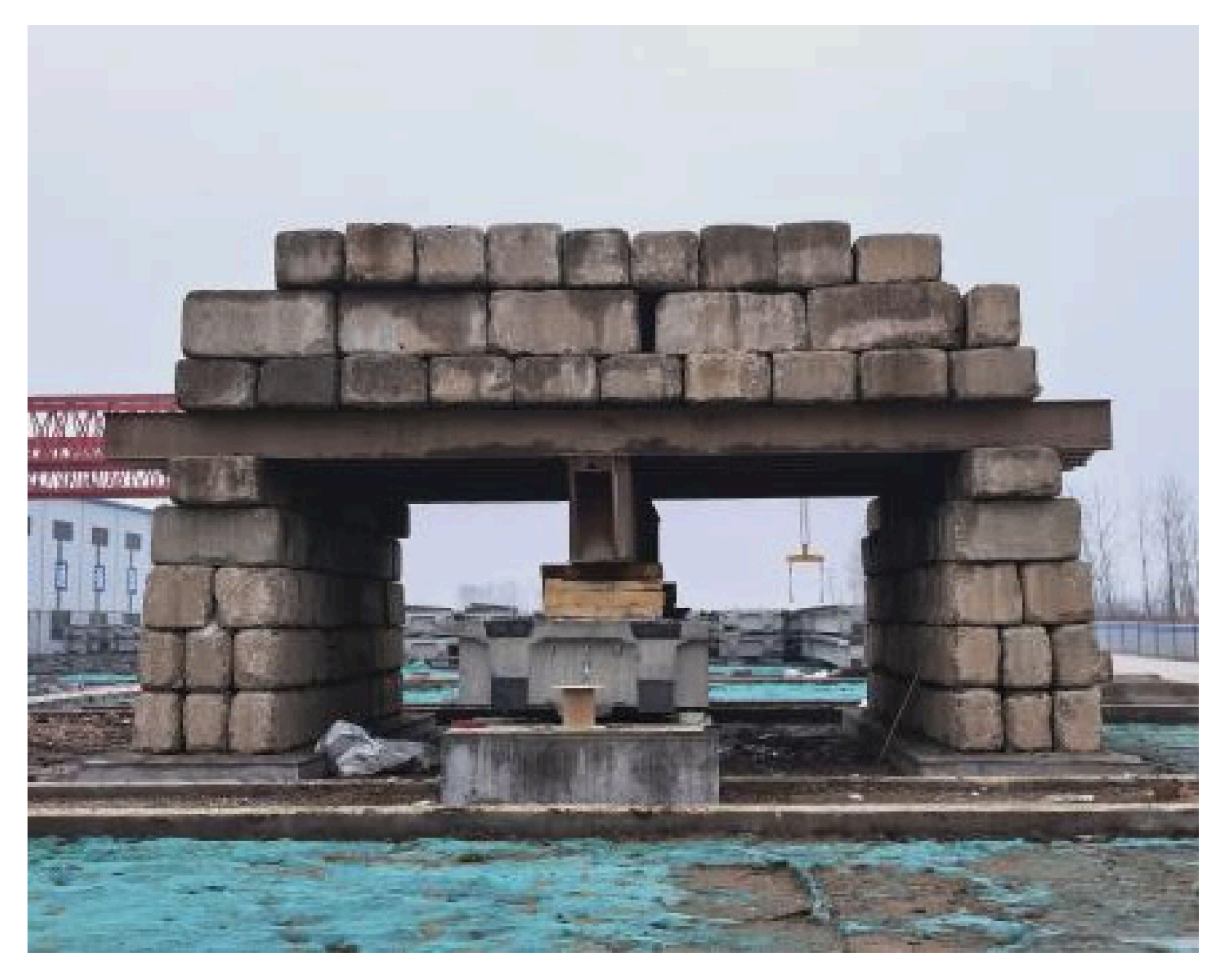
Reaction platform.

The test beam was selected from the first span of the first section of the Changshengkou Special Bridge. The beam is 16 m long and is a prestressed concrete π-shaped beam fabricated by the pre-tensioning method. The concrete strength grade is C50 and it belongs to Class A prestressed concrete components. The test adopted a single-point progressive loading method at the mid-span to conduct a destructive static bending test. The loading equipment was a 500T hydraulic jack, and the reaction system used concrete counterweights for loading. The total counterweight was no less than 310 tons; the reaction platform is shown in Figure 2.

### 3.2. Finite Element Model and Structural Force Calculation

To determine the test loading control internal forces and the impact coefficient, a finite element model of the 16 m 2# π-beam was established using Midas Civil. The main girder was simulated with spatial beam elements, and the entire bridge was discretized into 18 nodes and 19 elements, with a calculated span of 15.16 m. The boundary conditions were set to mimic actual simply supported bearings: the left end node was restrained in longitudinal, transverse, and vertical translations (DX, DY, DZ), while the right end node was restrained only in transverse and vertical translations (DY, DZ), with longitudinal translation released; rotational degrees of freedom were unrestrained at both ends, consistent with the simply supported beam behavior.

Material parameters were adopted from the design documents. The main girder and wet joints used C50 concrete with elastic modulus E_c_ = 3.45 × 10^4^ MPa, Poisson’s ratio 0.2, and unit weight 26.0 kN/m^3^. The prestressing tendons were low-relaxation high-strength steel strands with nominal diameter 15.2 mm, tensile strength standard value f_pk_ = 1860 MPa, elastic modulus E_p_ = 1.95 × 10^5^ MPa, control tensile stress σ_con_ = 1358 MPa, and relaxation coefficient 0.3. Section properties were input according to the actual geometric dimensions of the π-beam from the design drawings, and the program automatically computed the area, moment of inertia, and centroid position. Prestress effects were modeled as a pretensioned member, with effective prestress applied as equivalent nodal loads, and all code-specified prestress losses were taken into account.

The following load cases were considered in the model:

1. Dead load stage I, including self-weight of the bare beam and wet joint load (applied as a uniform line load of 0.4 × 0.18 × 26 = 1.872 kN/m);
2. Dead load stage II, including the cast-in-place concrete overlay (0.1 m thick, unit weight 26 kN/m^3^), asphalt pavement (0.1 m thick, unit weight 24 kN/m^3^), and barrier load (distributed to the 2# beam via the transverse distribution coefficient, with a value of 3.086 kN/m);
3. Live load, using Highway-I grade, arranged in the most unfavorable number of lanes (three lanes), with the impact factor included. The transverse distribution coefficients for vehicle loads were obtained by the grillage method; the 2# middle beam had the largest distribution coefficient (0.616) under three-lane loading, and was therefore selected as the most unfavorable test beam. The transverse distribution coefficient for the barrier load was taken as 0.140.

Modal analysis yielded a first-order vertical natural frequency of 6.312 Hz and a corresponding natural period of 0.158 s, based on which the impact factor for vehicle loads was calculated according to the code, providing a basis for test loading. The model gave a mid-span bending moment of 3527.5 kN·m for the basic combination at the ultimate limit state. The design flexural resistance of the normal section, checked against the Design Specifications for Highway Reinforced Concrete and Prestressed Concrete Bridges and Culverts (JTG 3362-2018 [25]), was 3706.2 kN·m. Since the test was conducted on the bare beam, the self-weight mid-span moment (778.4 kN·m) already existed; therefore, the target test loading internal forces were taken as the basic-combination corresponding value:

(7) 3527.5−778.4=2749.1 3527.5−778.4=2749.1 kN·m

the design-resistance corresponding value:

(8) 3706.2−778.4=2927.8 3706.2−778.4=2927.8 kN·m.

The latter was adopted as the maximum test load P1, and a step-by-step loading scheme from 0.3P1 to 1.9P1 was formulated accordingly, as shown in Table 2, ensuring that the test covered both the serviceability and ultimate limit states.

The modeling approach and calculation results fully support the determination of test load steps, sensor layout, and failure control criteria, ensuring the scientific validity and safety of the static load test. This finite element model not only provided the impact factor and internal force control values, but also served as a benchmark for subsequent theoretical calculations of deflection and strain, as well as for the evaluation of verification coefficients.

### 3.3. Loading Scheme

This test was conducted on the girder fabrication platform, and the test method employed the single-point loading method. The single-point loading method involves applying force to the girder using a jack. The loading device mainly consists of a jack, a reaction device (pile loading method), and a force-measuring instrument. During the test, a 500T jack was used at the loading point on the mid-span surface of the girder to perform single-point progressive loading of the girder, with strict control over the magnitude of each load level. Concrete test blocks were used as counterweights for the reaction system.

The test is carried out with staged loading, at a slow and uniform speed, with the loading and unloading rates not exceeding 3 kN/s. The loading time intervals meet the requirements for the stability of the structural response. The holding time after each level of loading is no less than 5 min. After holding for 5 min, the deflection (strain) of the precast beam is measured. Data is collected every 2 min. When the increment of the two consecutive measurements is less than 3 times the resolution of the measuring instrument or 1% of the theoretical value, the test result of this level is recorded and the next level of loading is carried out.

### 3.4. Measurement Point Layout

#### 3.4.1. Strain Measurement Points

**Figure 3:**
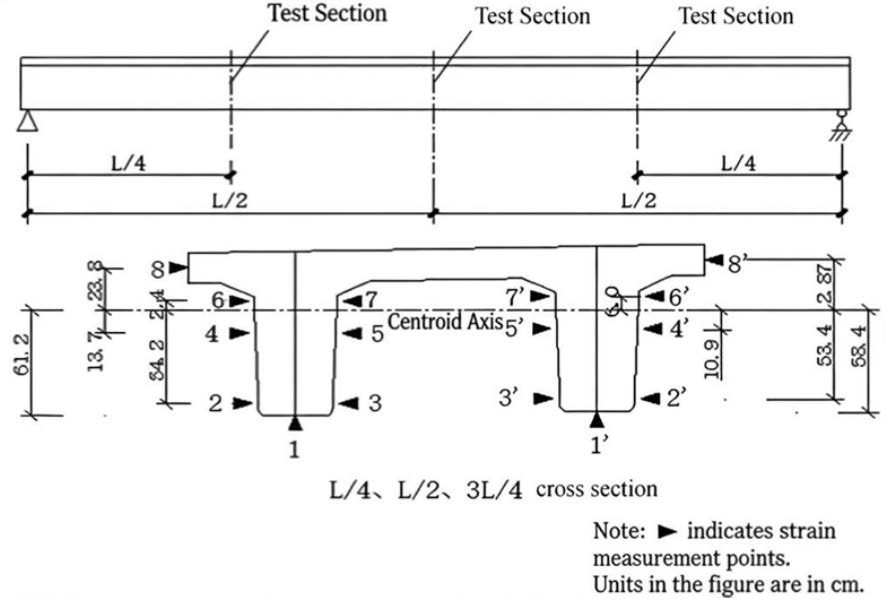
Layout of strain gauges.

**Figure 4:**
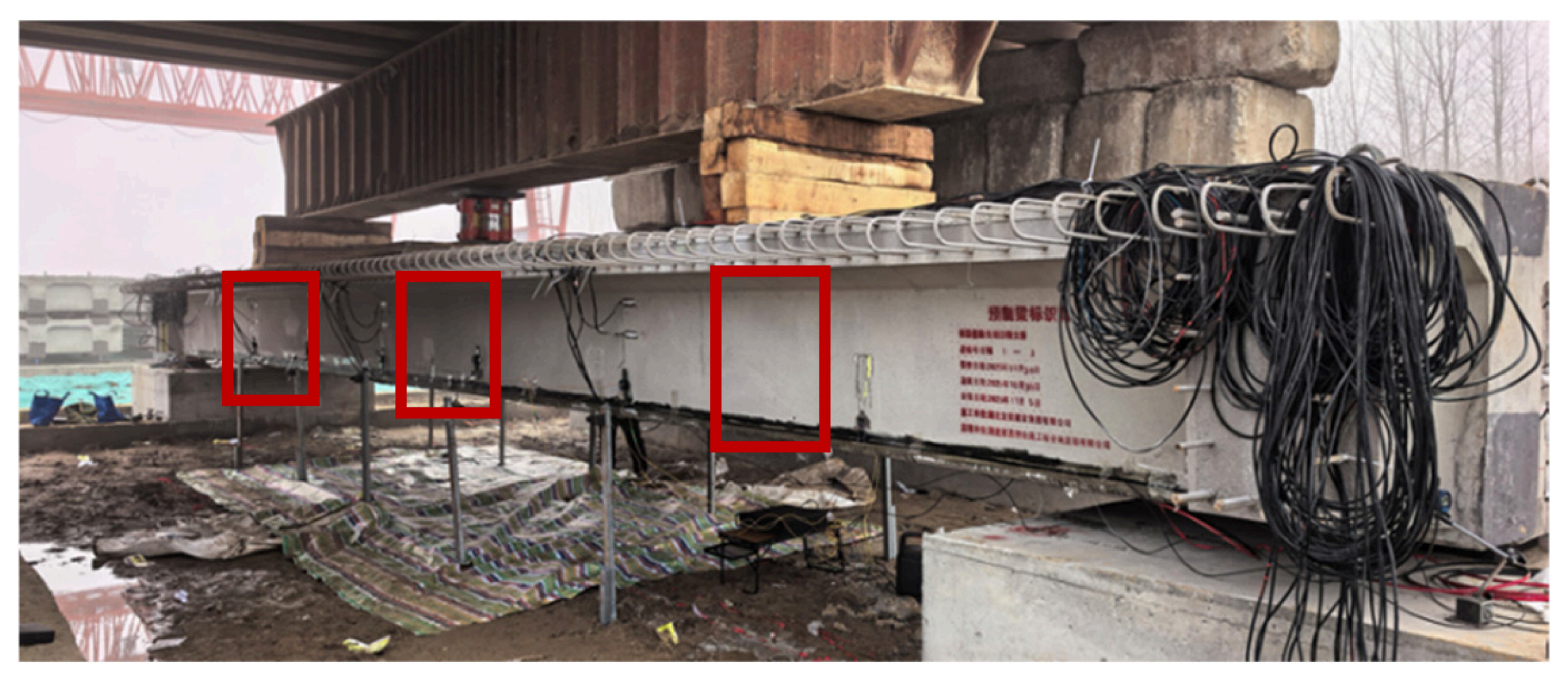
Photograph of strain gauge installation.

**Figure 5:**
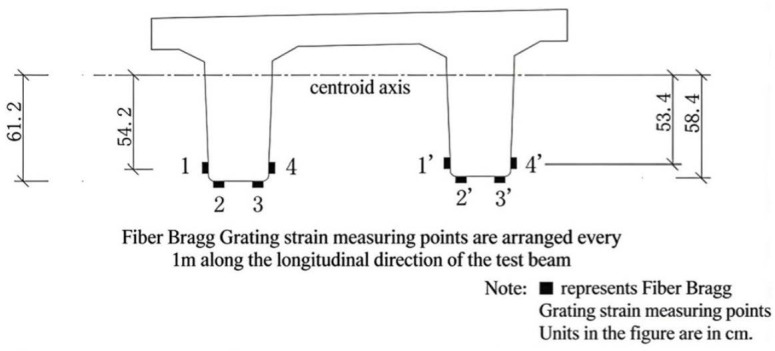
Layout of the UWFBG array strain cables.

**Figure 6:**
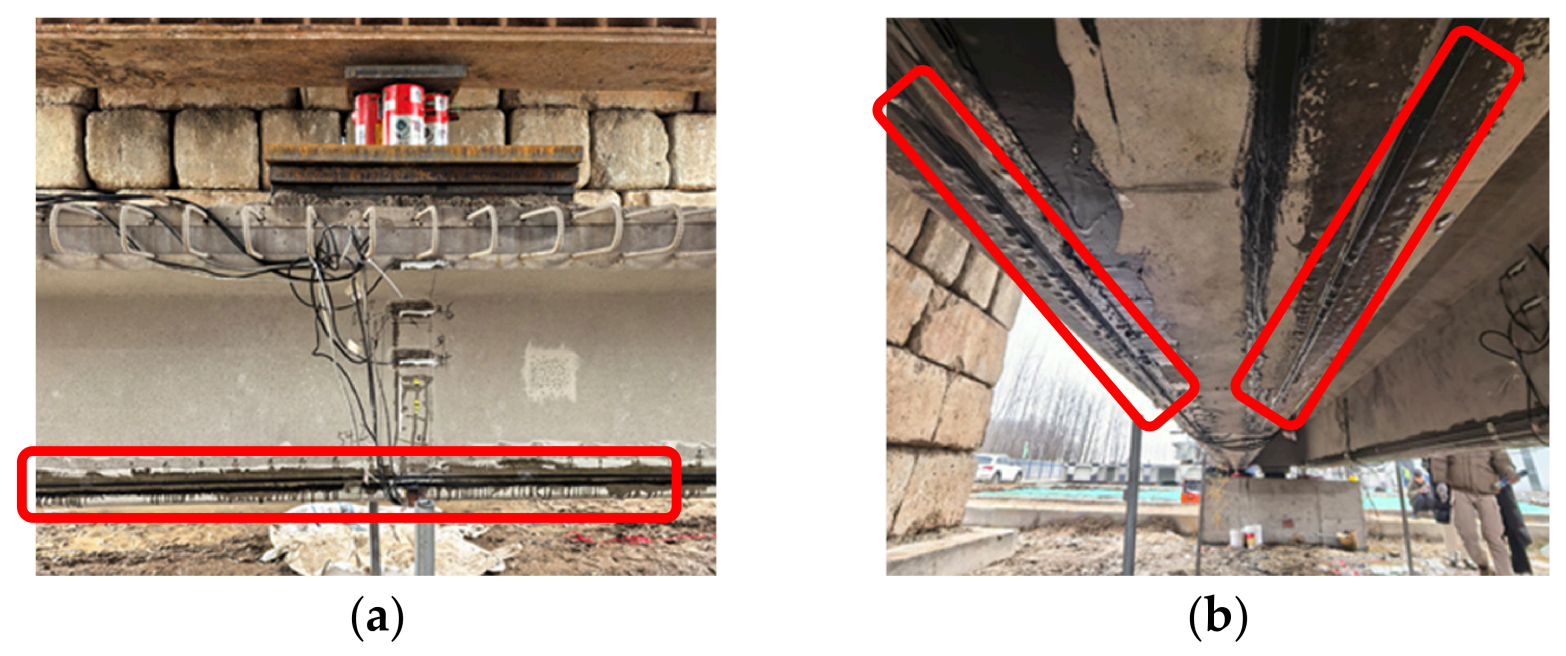
Optical cable installation diagram. (**a**) Web; (**b**) beam plate.

In this experiment, traditional point-type resistive strain gauges and grating array strain cables were used for strain measurement. The layout and on-site arrangement of the strain measurement points are shown in Figure 3, Figure 4, Figure 5 and Figure 6.

The point-type strain gauges were respectively arranged at the 1/4 section, mid-span section, 3/4 section, and both sides of the support section of the beam, with 6 measurement points on each section, totaling 30 measurement points, to achieve discrete point strain monitoring at key sections.

In contrast to the installation of traditional point-type strain gauges, the grating array strain-sensing optical cable, with a total length of 163 m, is laid along the entire span of the beam. The surface-mount method is adopted for deployment, utilizing Hummer-brand epoxy-impregnated structural adhesive to ensure full coordinated deformation between the cable and the beam. Two cables are installed on each side (inner and outer) of the web, positioned 5 cm from the lower edge of the bottom plate; four cables are symmetrically arranged along the bottom plate, spaced 5 cm from the edges of the web, resulting in a total of eight cables to achieve comprehensive continuous strain monitoring across the entire beam. The grating array cables employed in this experiment have a grating pitch of 1 m, a reflectivity of approximately 0.01%, and a central wavelength of about 1550 nm. A four-channel grating signal demodulator was used, with a signal sampling frequency of 10 Hz.

#### 3.4.2. Crack Monitoring Points

**Figure 7:**
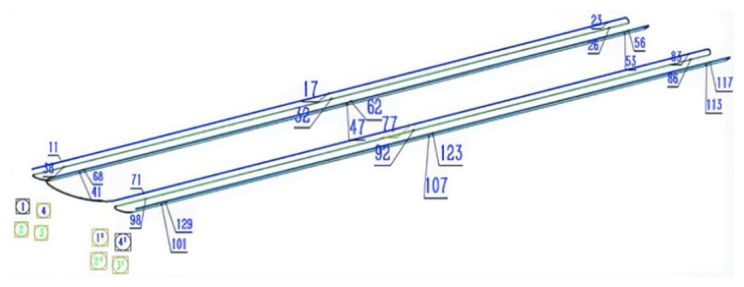
Positioning schematic of the UWFBG Array Strain Sensing cable.

Monitoring the occurrence of cracks at the most unfavorable stress position of the π beam (the bottom area of the mid-span of the beam) means that the strain sensing cables of the grating array with ID numbers 17, 32, 47, 62, 77, 92, 107, and 123 are the measurement points for cracks. The layout and positioning of the optical cable are shown in Figure 7 and Table 3.

Crack marking method:

1. For initial cracks (surface shrinkage cracks and surface damage cracks), use a blue pencil for marking. Before loading, use a 10× magnifying glass to carefully observe the surface of the beam and look for cracks. Draw a mark about 1 mm to the right of the crack in the direction of the crack, and mark the ends with a crossbar to distinguish whether the crack extends after loading.
2. For stress cracks, use a red pencil for annotation. Outline the direction of the crack about 1 mm to the right and seal the ends with a crossbar, and indicate the starting load level.

Crack observation time:

3. Before loading, carefully observe the initial cracks and local defects at the specified parts of the beam. Mark them with the specified color pencil.
4. After loading, during each load-holding period, carefully check if cracks or initial cracks extend at the lower edge, flange, and bottom of the beam. If cracks or initial cracks extend, annotate them with a red pencil and indicate the load level; measure the crack width.

#### 3.4.3. UWFBG Array System Specifications

The UWFBG array sensing system employed in this experiment consists of eight parallel optical cables. The key specifications of the system are summarized in Table 4, and detailed descriptions of the critical parameters are provided below.

The eight optical cables were connected to the four-channel interrogator (FL-S-3000, Fengli Optoelectronics Technology Co., Ltd., Wuhan, China) via time-division multiplexing: two cables were serially connected on each channel with low-loss optical connectors. With the grating reflectivity of approximately 0.01%, the total insertion loss of each 30-grating chain is within an acceptable range, and no signal crosstalk is observed. The nominal strain resolution, calculated from the 1.0 pm wavelength resolution and 1.2 pm/με strain sensitivity coefficient, is approximately 0.83 με. Factory calibration confirmed that all channels feature an SNR higher than 20 dB, allowing stable spectral peak tracking throughout the test.

The optical cables are laid in a winding manner along the full length of the beam, with one cable deployed on the inner and outer sides of each web and two cables symmetrically arranged on the bottom slab with a 5 cm offset from the web edges, resulting in a total of eight optical cables for full-domain continuous strain monitoring. Each optical cable contains 15 gratings with a nominal grating pitch of 1.0 m and a gauge length of 1.0 m per sensing unit, corresponding to a spatial resolution of 1.0 m. The reflectivity of each ultra-weak grating is approximately 0.01%, which enables dense wavelength-division multiplexing and time-division multiplexing on a single fiber while maintaining low insertion loss over long distances, allowing for the multiplexing of thousands of measurement points on a single optical fiber.

The optical signals are interrogated by a commercial four-channel high-speed demodulator (model FL-S-3000), which provides a wavelength resolution of 1.0 pm. In this test, the sampling frequency was set to 10 Hz to balance data volume and real-time response, which is sufficient to capture the quasi-static loading process. The central wavelength of the gratings is approximately 1550 nm, and the system’s strain measurement range is ±8000 με, as validated during the destructive test where the actual strain values reached up to ~8000 με while maintaining signal continuity, owing to the robust packaging and the inherent strain tolerance of the optical fiber.

Calibration procedure: The calibration of the UWFBG array system was performed using the dedicated management software. After connecting the demodulator to the local network and powering on the system, the software was launched to establish communication with the instrument. The sensing channel connected to the optical cable was selected, and the “Channel Calibration” function was activated. For each selected channel, the grating type was specified according to the actual sensor configuration: vibration gratings were designated as the chirped type, while strain and temperature measurement gratings were selected as either single-wavelength or dual-wavelength types based on their specific design. Upon completion of the calibration, the software automatically displayed the number of gratings identified, which, combined with the known grating spacing, allowed for the calculation of the total sensing cable length and a preliminary assessment of link integrity.

To ensure the accuracy and reliability of the calibration, the optical intensity curve was carefully monitored. If the maximum value of the intensity curve fell below 5000, the optical gain was increased, and the calibration was repeated to avoid missing gratings due to insufficient signal strength. Conversely, if the intensity curve approached saturation, the gain was reduced, and recalibration was performed. Following successful calibration, the sampling frequency was configured via the software’s frequency setting interface. The system automatically calculated the maximum allowable frequency based on the wavelength distribution and total length of the sensing cable, and the user-defined frequency was required not to exceed this limit. Notably, the frequency setting needed to be re-applied after each channel calibration.

## 4. Test Results and Analysis

### 4.1. Law of Crack Initiation and Propagation

**Figure 8:**
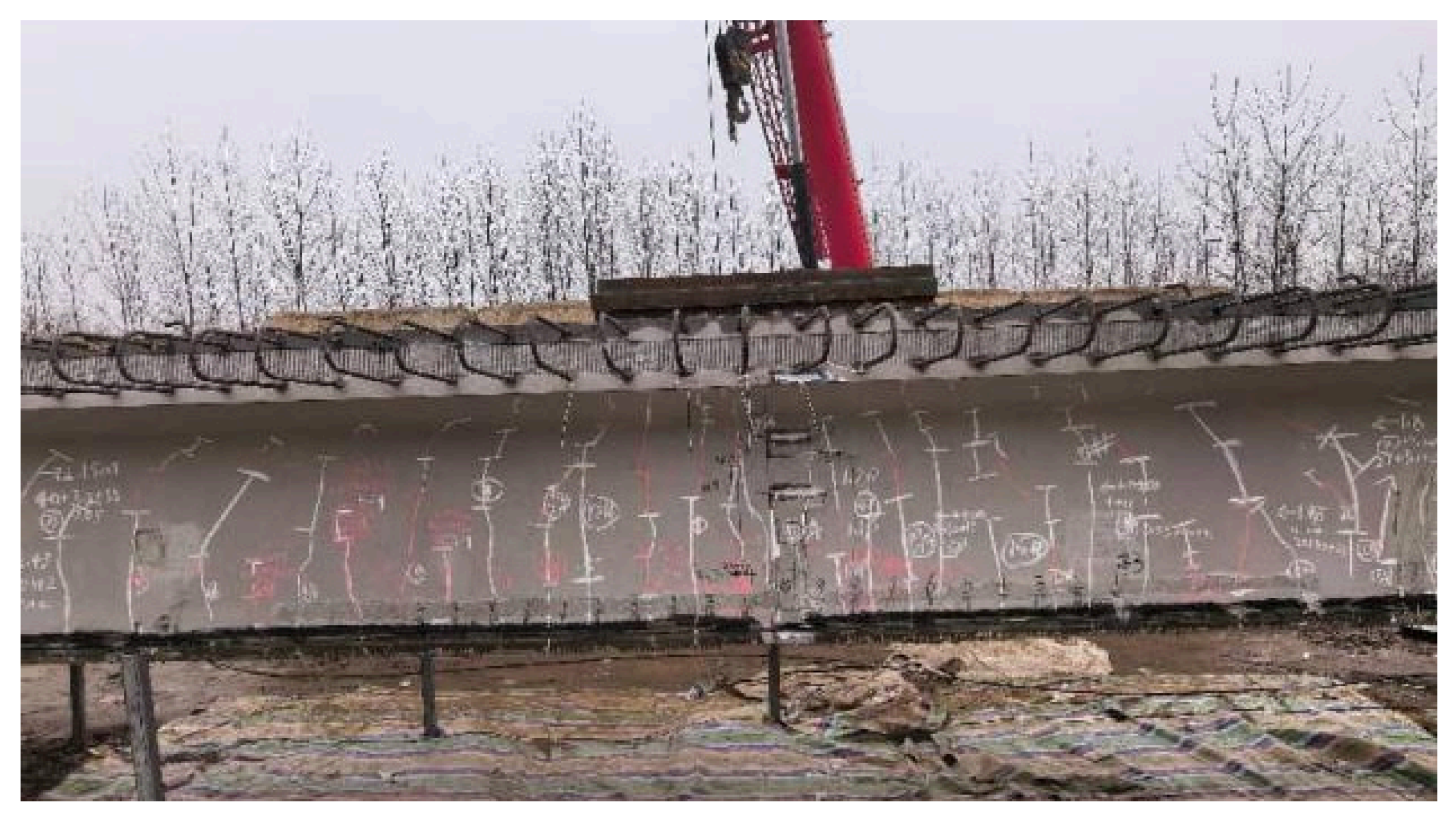
On-site crack recording chart.

After the loading test, data were collected once the structural response stabilized. At the same time, the development of cracks was observed and recorded. During the loading process, as shown in Figure 8, the initial cracks were marked with a blue pencil, and the newly generated cracks due to the force were marked with a red pencil. The positions, lengths, widths and expansion states of the cracks will be recorded.

**Figure 9:**
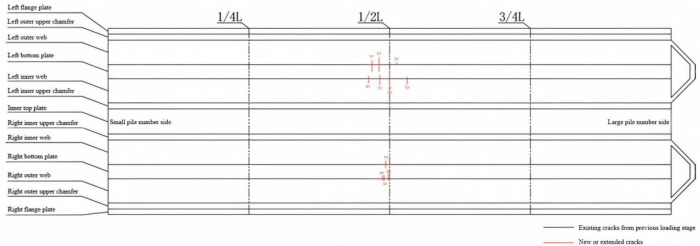
Overall schematic diagram of the crack, with a force of 848.7 kN.

**Figure 10:**
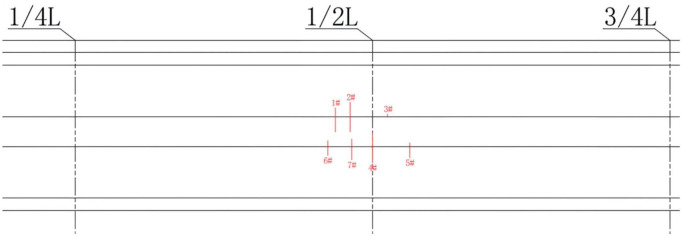
848.7 kN-Schematic diagram of the left crack.

Table 5 displays the left crack condition under the load of 848.7 kN (0.9P1).Based on the monitoring data of artificial cracks, the evolution of cracks in the π-shaped beam shows a distinct phased characteristic:

The stage of crack initiation: As shown in Figure 9 and Figure 10, when the load reached 848.7 kN (0.9P1), cracks began to appear in the mid-span web and bottom plate of the beam. A total of 11 new cracks emerged, with the maximum length being 40 cm and the maximum width 0.11 mm. The cracks were mainly of the L-type and were concentrated in the 1/2L area at the mid-span.

Crack expansion stage: During the process of the load increasing from 1.0P1 to 1.9P1, the number, length and width of cracks continued to increase. The crack type gradually evolved from L-shaped to U-shaped and penetrated throughout the entire span from 1/4L to 3/4L of the stressed area.

**Figure 11:**
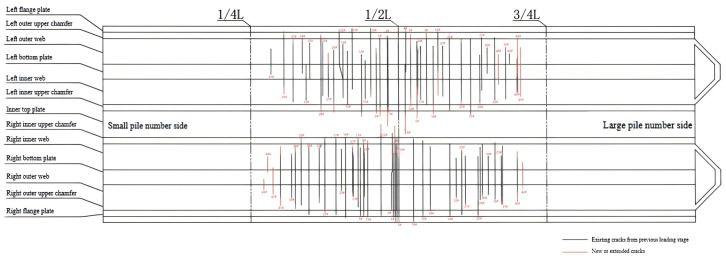
Overall schematic diagram of the crack, with a force of 1791.7 kN.

**Figure 12:**
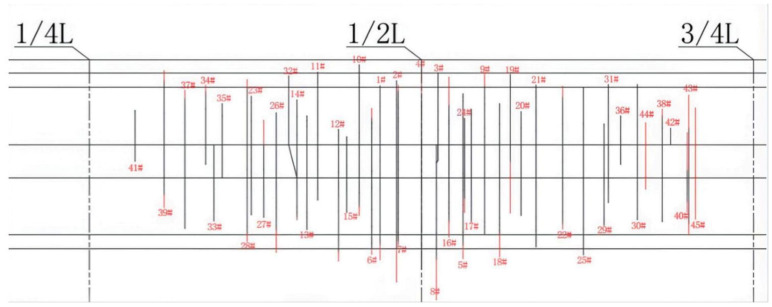
Schematic diagram of the left crack at 1791.7 kN.

**Figure 13:**
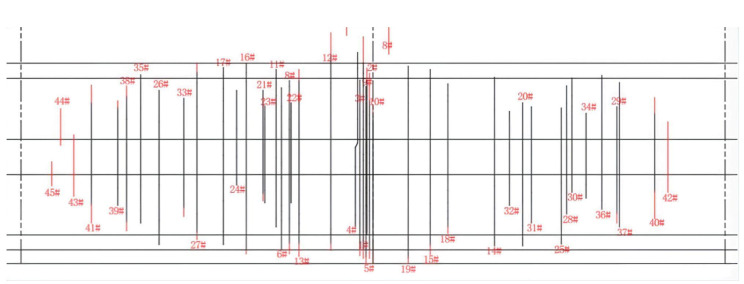
Schematic diagram of the crack on the right side (1791.7 kN).

Crack ultimate cracking stage: As shown in Figure 11, Figure 12 and Figure 13 (Note: Straight lines indicate the approximate paths and lengths of cracks for schematic illustration; actual crack trajectories are irregular and can be observed from the field photographs), when the load reached 1791.7 kN, the beam had accumulated 90 cracks, with the maximum crack width being 1.21 mm and the maximum length being 246.5 cm. The cracks penetrated the web and the bottom plate, and the structure entered a state of full-scale cracking.

To provide a clear overview of the crack evolution process from initiation to final failure, the essential parameters at each typical stage are summarized in Table 6. The initiation stage at 848.7 kN (0.9P1) is characterized by 11 newly developed L-shaped cracks, with a maximum width of 0.11 mm and a maximum length of 40 cm. During the expansion stage (from 1.0P1 to 1.9P1), the number, width, and length of cracks increased progressively, while the crack morphology gradually evolved from L-shaped to U-shaped and finally to penetrating cracks. At the ultimate cracking stage under 1791.7 kN (1.9P1), the total number of cracks reached 90, with a maximum width of 1.21 mm and a maximum length of 246.5 cm; the dominant crack type was U-shaped and fully penetrating through the web and bottom plate.

### 4.2. Strain Response Characteristics of the Grating Array

#### 4.2.1. Distribution and Evolution Characteristics of Fully Distributed Strain Space

**Figure 14:**
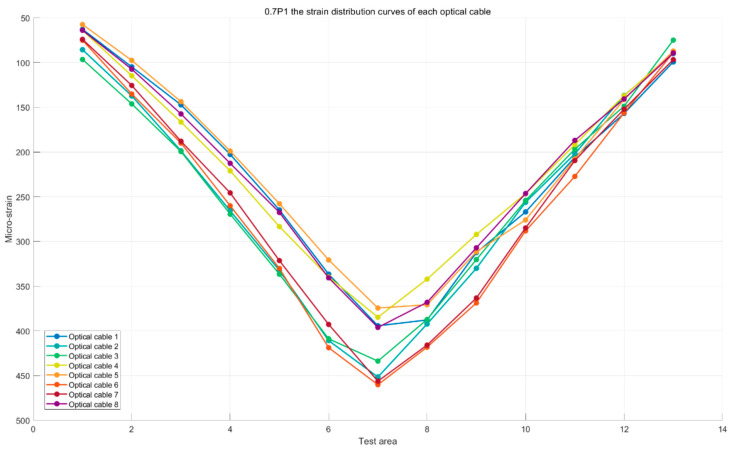
Strain distribution of the main girder measured by eight sensing cables.

As can be seen from the Figure 14, the strain distribution of the grating array strain sensing cable presents a typical symmetrical triangular-shaped distribution: the strain amplitude at the corresponding positions across the center of each cable (i.e., the crack measurement points ID17, 32, 47, 62, 77, 92, 107, and 123 in the previous text) is the largest, and it gradually decreases approximately linearly along the cable length towards the support areas at both ends of the beam, eventually approaching 0. This distribution completely conforms to the positive strain distribution law of a simply supported bending member, that is, the maximum bending moment occurs at the mid-span, the cumulative tensile strain is the most significant, the support bending moment is 0, and the strain approaches 0. This verifies the mechanical rationality of the strain monitoring data from the grating array.

**Figure 15:**
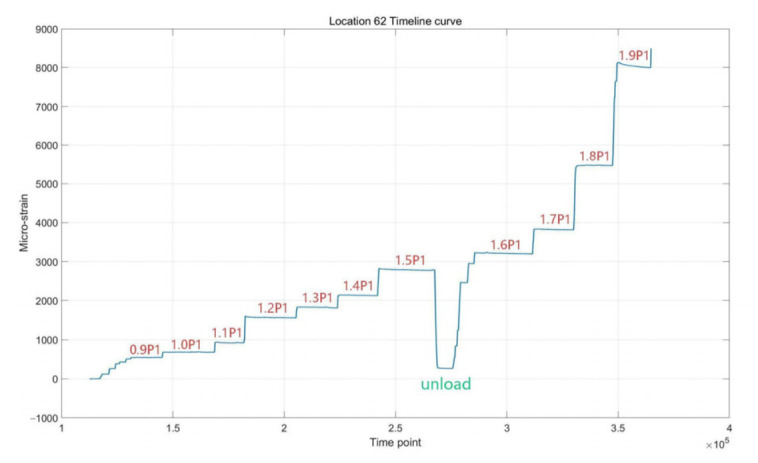
Time–history curve of strain at the grating measurement points in the mid-span area.

Figure 15 shows the time history curve of the strain at the grating measurement points in the mid-span area. It can be seen that the strain at the grating measurement points shows a significant response as the load level increases. There is a good correlation between the load and the strain, which confirms that the grating strain sensing cable can effectively reflect the force change trend of the structure. At the same time, it can clearly reflect the continuous evolution process of the structure from the elastic stage to the elastoplastic stage and, in real time, present the internal force distribution state of the entire beam. Moreover, even after the beam enters the cracking stage under the 0.9P1 load, it can still stably obtain the force evolution information that is difficult for finite element simulations to accurately characterize.

Meanwhile, the strain–time history curve exhibited a distinct stepwise upward trend throughout the entire graded loading process. After each load level was applied, the strain curve displayed stable stepwise growth; during the load maintenance phase, the strain values remained stable without significant abnormal fluctuations. In the later loading stages, the curve maintained a continuous and smooth growth trend, with no abrupt changes or instability, reflecting the continuous transition of the structure from the elastic stage to the elastoplastic stage. During the unloading phase, the strain at all measurement points decreased rapidly, with relatively low residual strain, further confirming the overall mechanical characteristics of the structure during the loading process.

#### 4.2.2. Characteristics of Single-Point Strain–Load Response Stages

**Figure 16:**
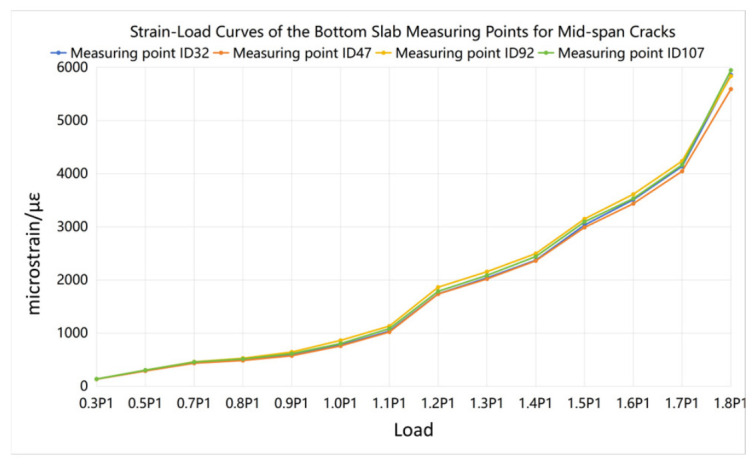
Strain–load curves of the bottom slab measuring points for mid-span cracks.

**Figure 17:**
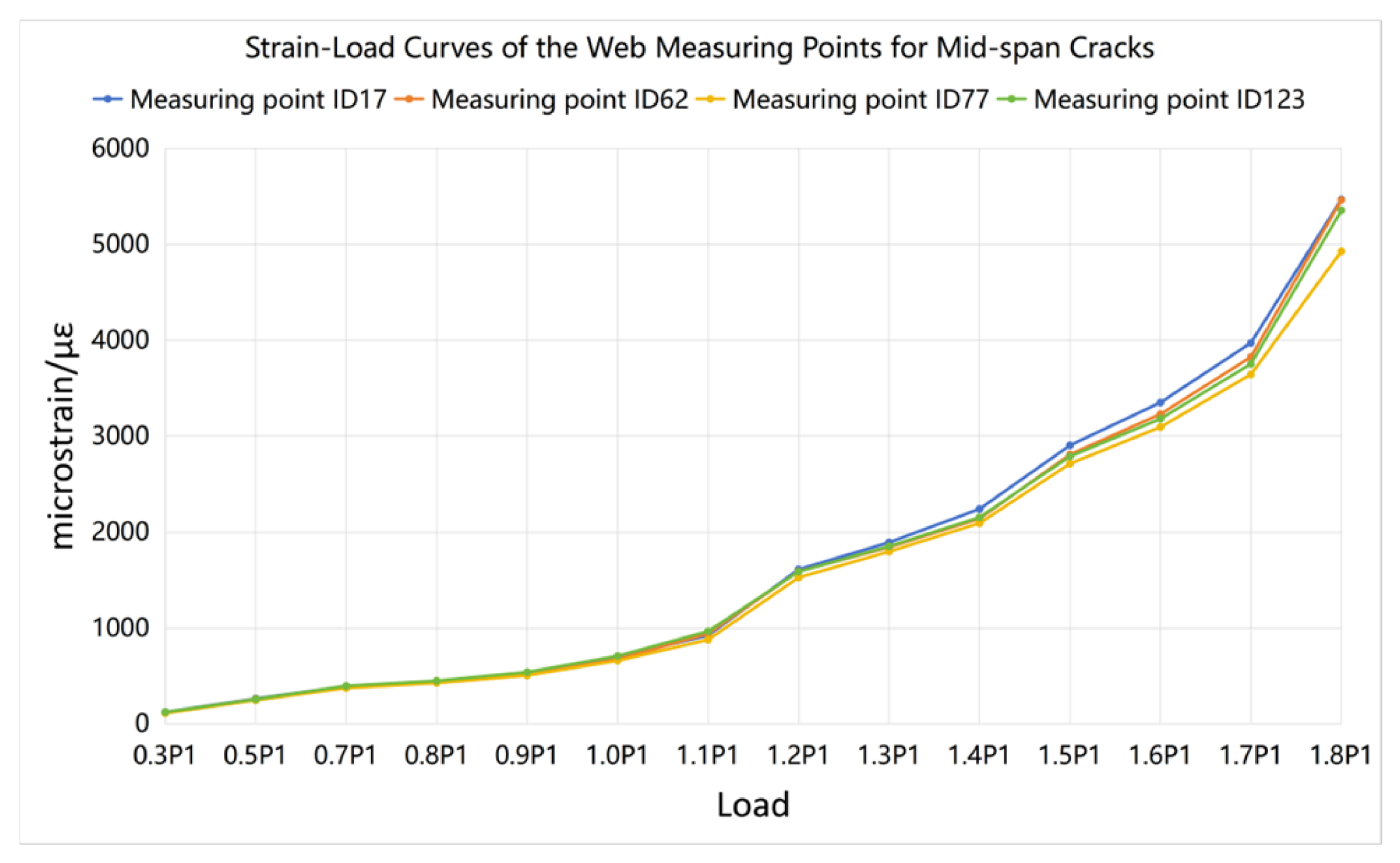
Strain-–load curves of the web measuring points for mid-span cracks.

Eight monitoring points for crack detection, namely ID17, 32, 47, 62, 77, 92, 107, and 123 of the grating array strain sensing cable, were selected. The strain–load relationship curves were plotted, and the elastic, crack initiation, crack propagation, plastic development, and complete failure of the π-shaped beam throughout the entire loading stage were compared. The strain response patterns of the grating were systematically analyzed. As shown in Figure 16 and Figure 17, the transformation from a linear to a nonlinear strain–load curve and the continuous increase in the curve slope are the core characteristics for identifying the evolution of cracks in grating monitoring. This can accurately reveal the entire evolution process and the force change mechanism of cracks from initiation and propagation to complete penetration.

1. Elastic stage (P < 848.7 kN)

During this stage, the beam is in an elastic state without cracking. The strain at each measuring point increases strictly linearly with the load, and the curve slope remains constant and unchanged. It is in perfect agreement with the theoretical calculation model of strain–load in the elastic stage. There is no sudden change in strain or curve inflection point at each measuring point. It directly reflects that the cross-sectional stress distribution of the beam is uniform, the overall stiffness is complete, the concrete has not reached the tensile strength, and there is no cracking phenomenon.

2. Crack initiation stage (P = 848.7 kN)

When the load reaches the critical load for crack initiation of 848.7 kN, the strain–load curves of the core force measurement points ID17 and ID32 at the mid-span first deviate from the linear relationship, showing a significant inflection point. The strain growth rate accelerates sharply, marking the first emergence of a stressed crack in the mid-span area. The other measurement points also show a slight nonlinear trend, and the curve slope begins to increase slightly. This feature is completely consistent with the observed L-shaped crack initiation position and time at the mid-span, verifying the precise positioning ability of the grating array for crack initiation.

3. Crack expansion stage (848.7 kN < P < 1791.7 kN)

As the load continues to increase, the cracks extend from the mid-span to the 1/4L and 3/4L areas. The shape gradually changes from L-shaped to U-shaped. The strain–load curves of the eight grating measurement points all show nonlinear rapid growth, and the curve slope continues to increase with the load. Among them, the slope of the mid-span ID17 and ID32 measurement points increases the most significantly, while the slope of the measurement points away from the mid-span, such as ID47, 62, 77, 92, 107, and 123, decreases successively. The change in slope amplitude is highly matched with the degree of crack development and the distance from the mid-span. It directly reflects the mechanical behavior of the beam’s stiffness continuously deteriorating and the cracks continuously expanding and extending.

4. Plastic development and complete failure stage (P ≥ 1791.7 kN)

After the load reaches 1791.7 kN, the beam enters the plastic development and overall cracking stage. The slope of the strain–load curves at each measuring point further increases, the load growth slows down or slightly recedes, and the strain still maintains a rapid growth trend. Until the concrete in the compression zone of the structure crushes and completely fails, the eight grating measurement points still continuously and stably output signals, completely recording the entire process of the beam’s plastic development and the complete penetration of cracks.

#### 4.2.3. Strain Field Distribution and Symmetry Verification

**Figure 18:**
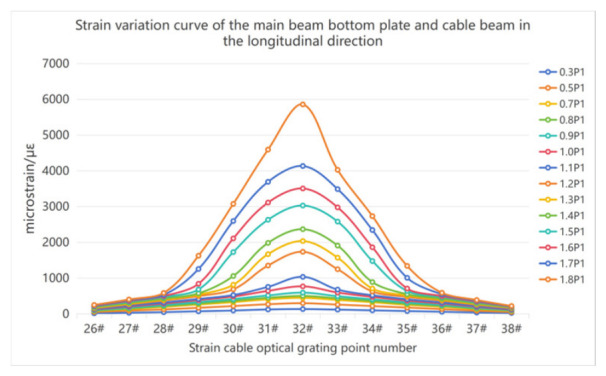
Strain variation curve of the main beam bottom plate and cable beam in the longitudinal direction.

**Figure 19:**
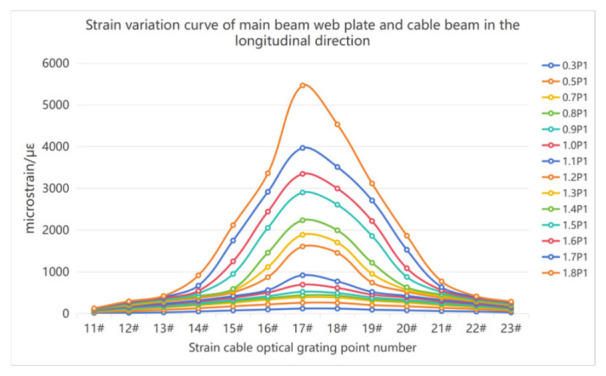
Strain variation curve of main beam web plate and cable beam in the longitudinal direction.

The strain distribution characteristics along the longitudinal direction of the main beam’s bottom plate and web plate under different loading stages are illustrated in Figure 18 and Figure 19, which present the strain variation curves measured by the distributed optical fiber sensing system. The grating array integrated into the optical cable beam enables continuous, real-time monitoring of the internal strain distribution along the entire length of the test beam, capturing the evolution of internal forces from the elastic loading stage to the crack initiation and propagation stage. Notably, even when the beam enters the crack formation stage under a load of 0.9P1 (where P1 represents the design cracking load of the specimen), the system maintains stable signal acquisition and reliable mechanical response data. This capability addresses a key limitation of conventional finite element simulations, which often struggle to accurately characterize the nonlinear strain evolution and local stress redistribution associated with crack initiation and propagation in concrete structures.

To further validate the reliability of the experimental data and the rationality of the loading scheme, a symmetry verification of strain data was conducted using symmetrical grating measurement points on the bottom plate and web plate. Under all tested loading conditions, the strain values obtained from the symmetrical measurement points exhibited a high degree of consistency, with negligible differences that fell within the allowable error range for structural monitoring. This excellent data consistency not only confirms the stability and precision of the distributed optical fiber sensing system in capturing full-field strain responses but also verifies the accuracy of the symmetrical loading method adopted in the test. The symmetrical distribution of strain observed in the curves further confirms the validity of the experimental data, as it aligns with the theoretical mechanical behavior of symmetrically loaded beams. Collectively, these results demonstrate that the experimental setup, including the loading scheme and monitoring system, is reliable and capable of providing accurate data for subsequent analysis of the structural performance of the test beam.

#### 4.2.4. Quantitative Correlation Analysis Between FBG Strain Peaks and Manual Crack Widths

To transform the crack monitoring capability from qualitative identification to quantitative evaluation, we performed a correlation analysis between the strain peaks recorded by the UWFBG array and the manually measured crack widths at corresponding locations. Since the grating array provides an average strain over a 1 m gauge length, a direct point-to-point comparison with individual crack widths is physically inappropriate. Instead, we adopted a segmental statistical approach: for each loading stage, the maximum strain value within the mid-span region (where cracking was most concentrated) was paired with the maximum crack width observed in the same region during the corresponding manual inspection.

**Figure 20:**
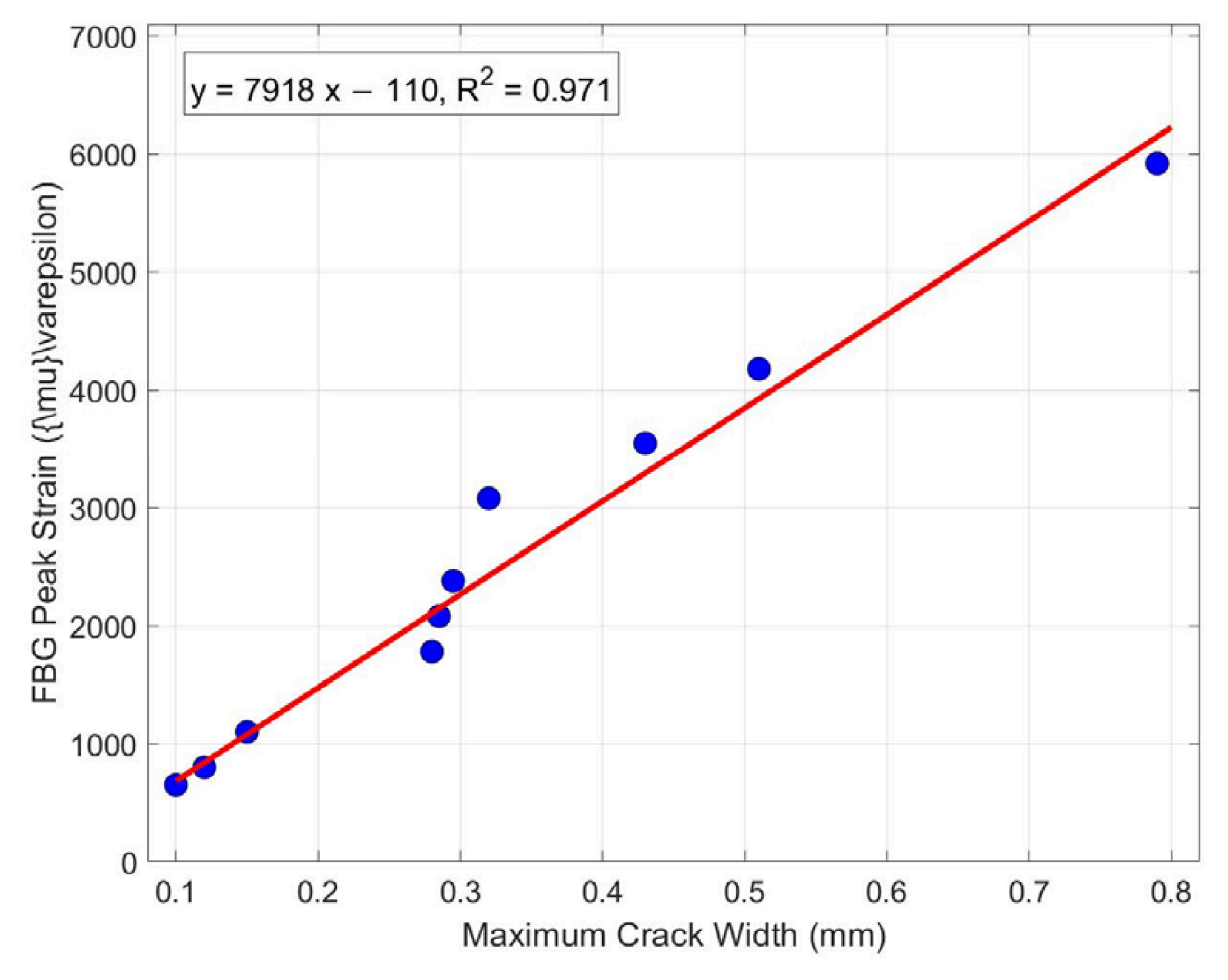
Correlation between FBG peak strain and maximum crack width under various loading levels (0.9P1 to 1.8P1).

Figure 20 presents the scatter plot of FBG peak strain versus maximum crack width under various load levels from crack initiation (0.9P1) to 1.8P1. A clear positive correlation is observed: as the crack width increases from 0.11 mm to 0.79 mm, the corresponding FBG peak strain rises from 597 με to 5860 με. Linear regression analysis yields a coefficient of determination (R^2^) of 0.971, with the fitting equation expressed as ε = 7918·w − 110 (where w is the crack width in mm and ε is the strain in με). This high R^2^ value indicates an excellent linear relationship between the two variables within the tested range, confirming that the FBG strain response can serve as a reliable quantitative indicator of crack severity.

It must be emphasized that this empirical correlation is strictly valid only for the tested π-beam under the specific test conditions. Factors such as concrete cover thickness, reinforcement ratio, prestress level, and sensor bonding quality can all influence the strain–crack width relationship. Consequently, the equation ε = 7918·w − 110 (R^2^ = 0.971) should not be directly applied to other structures. For any field deployment, a dedicated calibration procedure on the target structure is mandatory to obtain a reliable crack width estimation from FBG strain data.

#### 4.2.5. Analysis of the Effectiveness of UWFBG Array Monitoring

The comprehensive results obtained from the combined distributed and single-point monitoring systems provide robust validation of the performance of the grating array strain sensing system. Furthermore, the quantitative correlation analysis established in Section 4.2.4 demonstrates that the UWFBG strain peaks are linearly related to the manually measured crack widths, confirming that the system can provide not only qualitative spatial–temporal tracking but also quantitative severity assessment of cracks.

From the spatial dimension, the grating array enables continuous, full-field strain measurement along the entire length of the beam. As the applied load increases, the depth and slope of the triangular-shaped strain distribution curve exhibit a continuous and progressive increase. This characteristic evolution directly reveals the spatial expansion path of cracks, including their initiation location, propagation direction, and growth range, as well as the spatial degradation pattern of structural stiffness caused by crack formation and extension. Unlike conventional single-point monitoring methods, which can only capture discrete strain data at limited locations, the distributed grating array provides a complete strain profile that allows for the precise localization and tracking of crack-induced stress concentrations and stiffness reduction.

From the temporal dimension, the strain–load curves recorded by the grating array exhibit a distinct transformation from linear-to-nonlinear behavior, accompanied by a continuously increasing slope. This transition marks the critical time nodes of crack initiation, stable propagation, and unstable expansion, while the rate of change in the slope accurately quantifies the evolution rate of these processes. The high sampling frequency and stability of the grating array system ensure that even subtle changes in strain response during the early stages of crack formation are captured, providing a clear temporal signature of the structural transition from elastic to inelastic behavior.

The spatial and temporal information obtained from the grating array monitoring ise mutually consistent and complementary. Together, they fully demonstrate that the grating array strain sensing system can effectively reveal the entire force mechanism of the π-shaped beam throughout its service life, spanning from elastic operation, crack initiation, crack expansion, to the final plastic failure stage. The system is capable of accurately capturing both the time evolution pattern and spatial distribution characteristics of cracks, including their initiation, propagation, and impact on structural performance. These results confirm that the grating array sensing technology provides multi-dimensional, continuous and full-field reliable technical support for the structural health monitoring and safety assessment of large-span π-shaped beams, addressing key limitations of traditional monitoring methods in capturing the full picture of structural damage evolution. In particular, the empirical strain–crack width relationship obtained from this specimen (R^2^ = 0.971) illustrates the potential for quantitative crack severity estimation from distributed strain data; however, its generalization requires structure-specific calibration, as discussed in Section 4.2.4.

### 4.3. Comparison of Monitoring Performance Between Grating Arrays and Resistive Strain Gauges

It should be noted that theoretical strain values are derived from the elastic uncracked section (Equation (6)); therefore, quantitative accuracy assessment is performed strictly within the elastic stage (0.3 P1–0.9 P1). Beyond cracking (≥1.0 P1), the theoretical values are mere extrapolations that do not represent the actual structural strain. Consequently, the evaluation is separated into two distinct questions: (i) elastic-range measurement accuracy, and (ii) post-cracking sensor survivability and data continuity.

#### 4.3.1. Elastic-Range Measurement Accuracy (0.3 P1–0.9 P1)

Table 7 and Table 8 present the strain measurements of the UWFBG array and resistance strain gauges at the bottom plate and web plate, respectively, alongside the theoretical elastic values. Within the 0.3 P1–0.9 P1 range, both sensors are evaluated against the theoretical predictions to determine their measurement accuracy.

**Figure 21:**
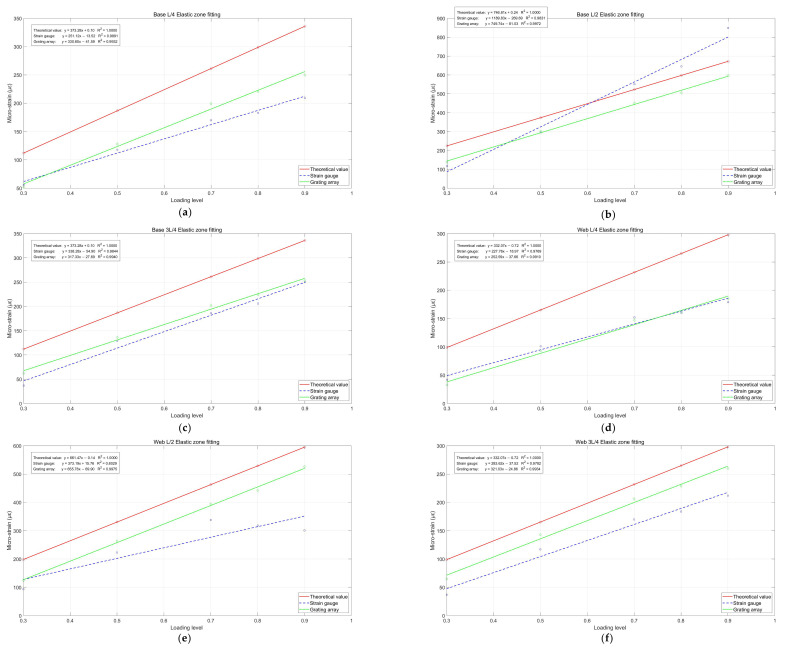
Linear strain–load fitting curves within the elastic loading stage (0.3 P1–0.9 P1) at six critical sections of the prestressed π-beam. Comparison among theoretical elastic strain, resistance strain gauge measurements, and UWFBG grating array responses. (**a**) Bottom plate L/4 section; (**b**) bottom plate L/2 mid-span section; (**c**) bottom plate 3L/4 section; (**d**) web plate L/4 section; (**e**) web plate L/2 mid-span section; (**f**) web plate 3L/4 section.

To quantify the linearity of the strain response, strain–load data from six key control sections (1/4 span, mid-span, and 3/4 span of both the bottom plate and web) were fitted using linear regression exclusively over the elastic loading stage (0.3 P1–0.9 P1), as shown in Figure 21. The coefficient of determination (R^2^) was calculated from these elastic-stage fits. Table 9 summarizes the resulting R^2^ values.

The fitting results in Table 9 demonstrate that the UWFBG array exhibits excellent linear correlation with the applied load in the elastic stage, with R^2^ values ranging from 0.9910 to 0.9975 across all six sections. In contrast, the resistance strain gauges yield R^2^ values between 0.8029 and 0.9891; in particular, the web mid-span (L/2) strain gauge shows a markedly lower linearity (R^2^ = 0.8029) owing to increased scatter at low strain levels. For both bottom plate and web plate sections, the grating array consistently outperforms the strain gauges, providing strain measurements that agree more closely with the theoretical elastic predictions.

**Figure 22:**
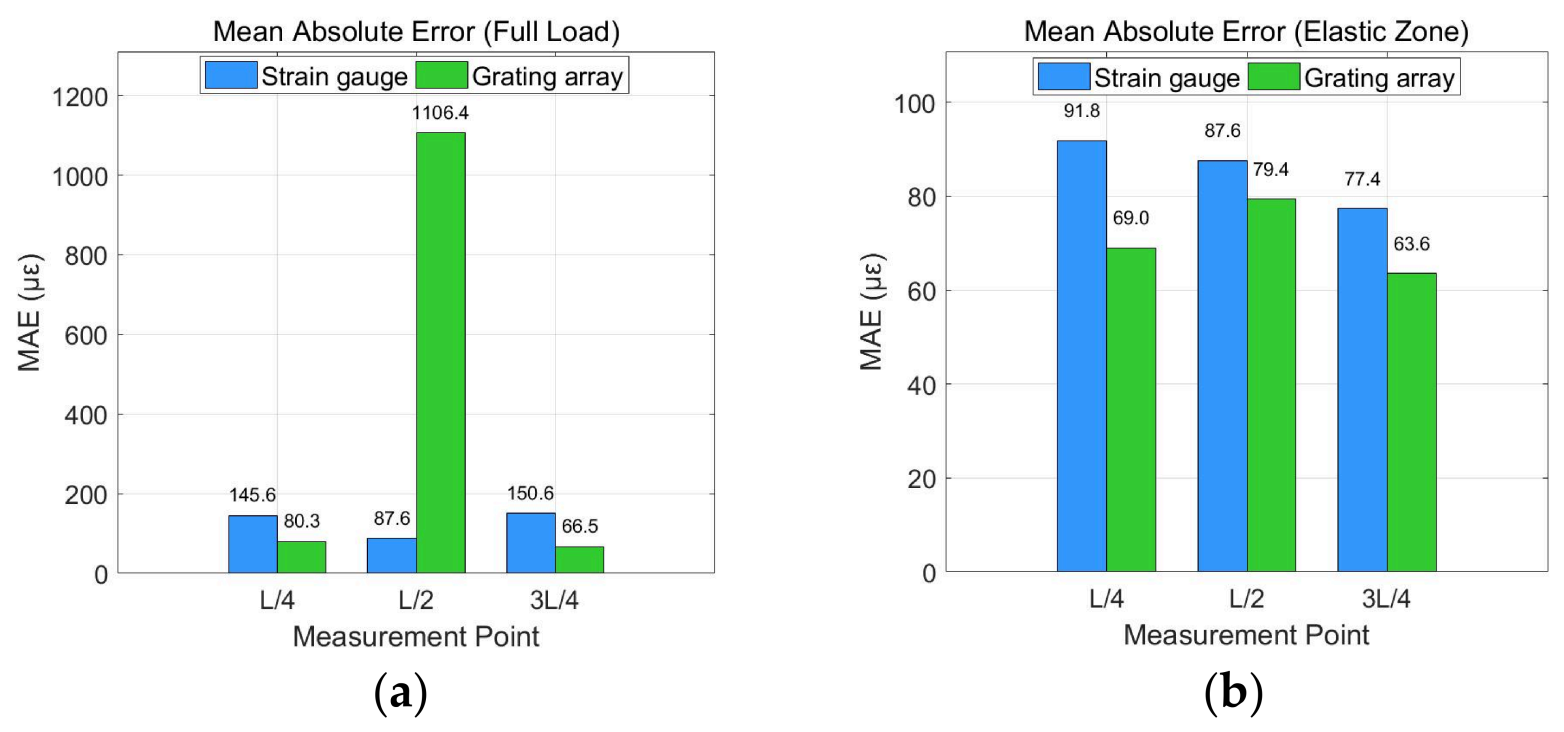
Comparison of Mean Absolute Error (MAE) between strain gauge and grating array under different loading stages: (**a**) full loading condition (0~1.8P1); (**b**) elastic zone condition (0.3~0.9P1).

**Figure 23:**
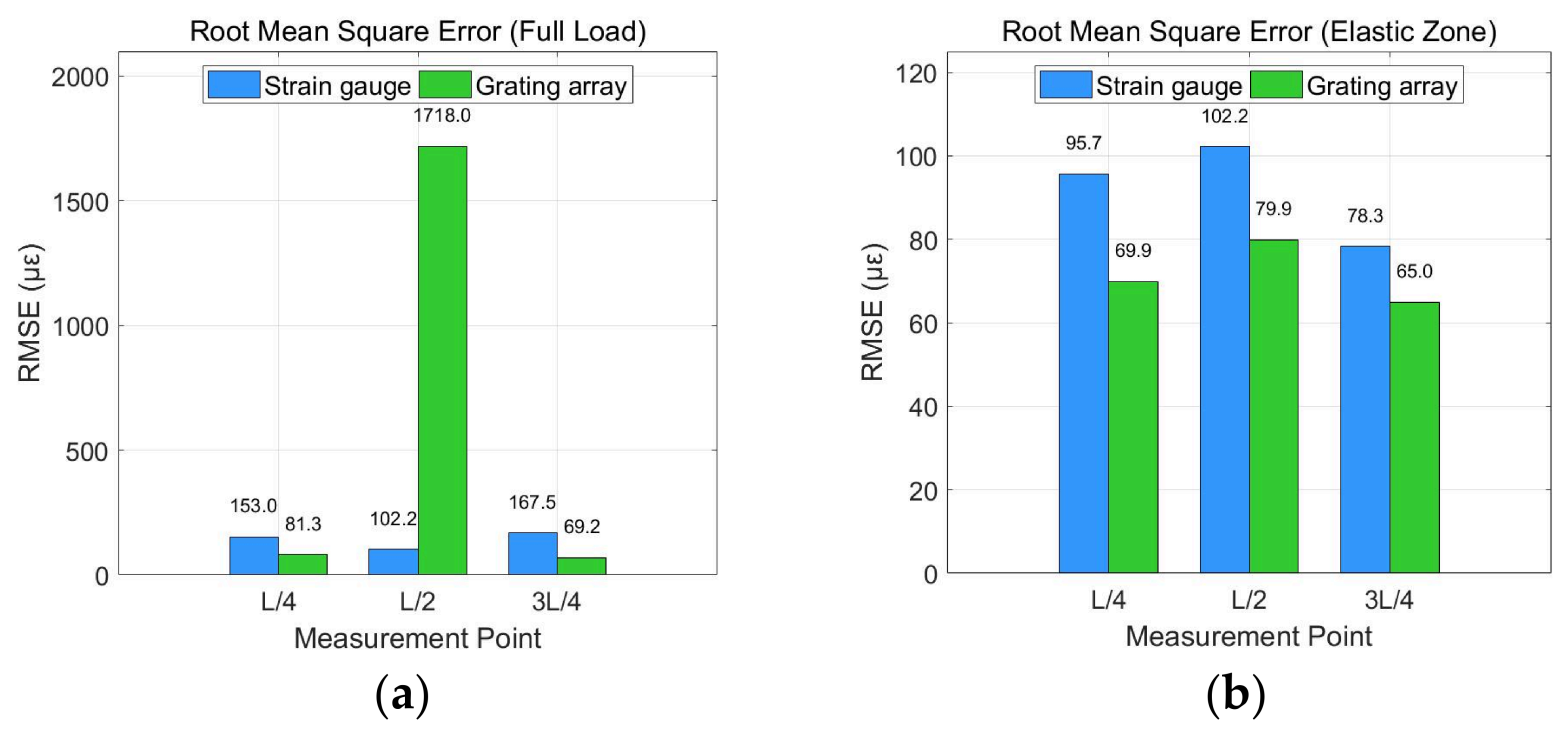
Comparison of Root Mean Square Error (RMSE) between strain gauge and grating array under different loading stages: (**a**) full loading condition (0~1.8P1); (**b**) elastic zone condition (0.3~0.9P1).

**Figure 24:**
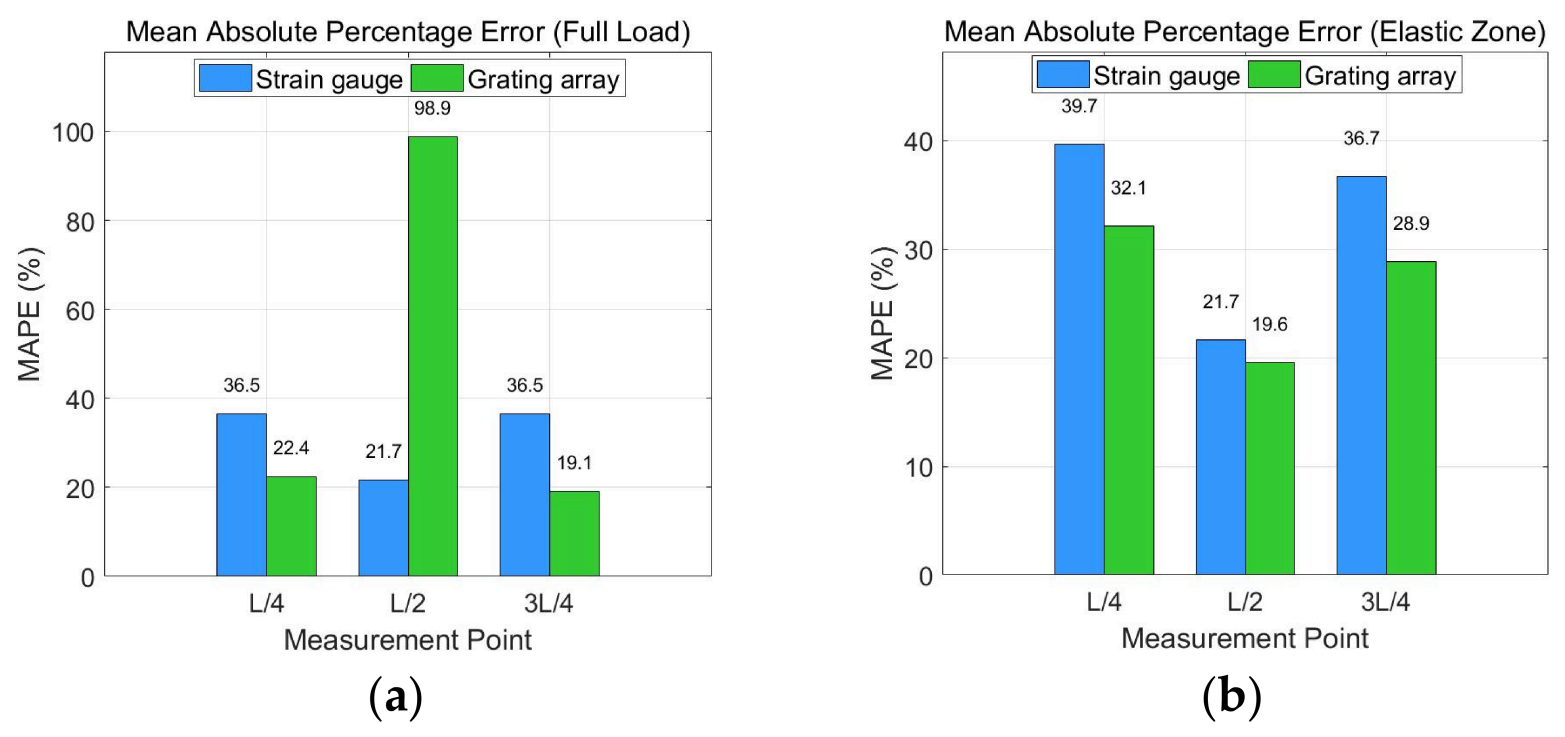
Comparison of Mean Absolute Percentage Error (MAPE) between strain gauge and grating array under different loading stages: (**a**) full loading condition (0~1.8P1); (**b**) elastic zone condition (0.3~0.9P1).

**Figure 25:**
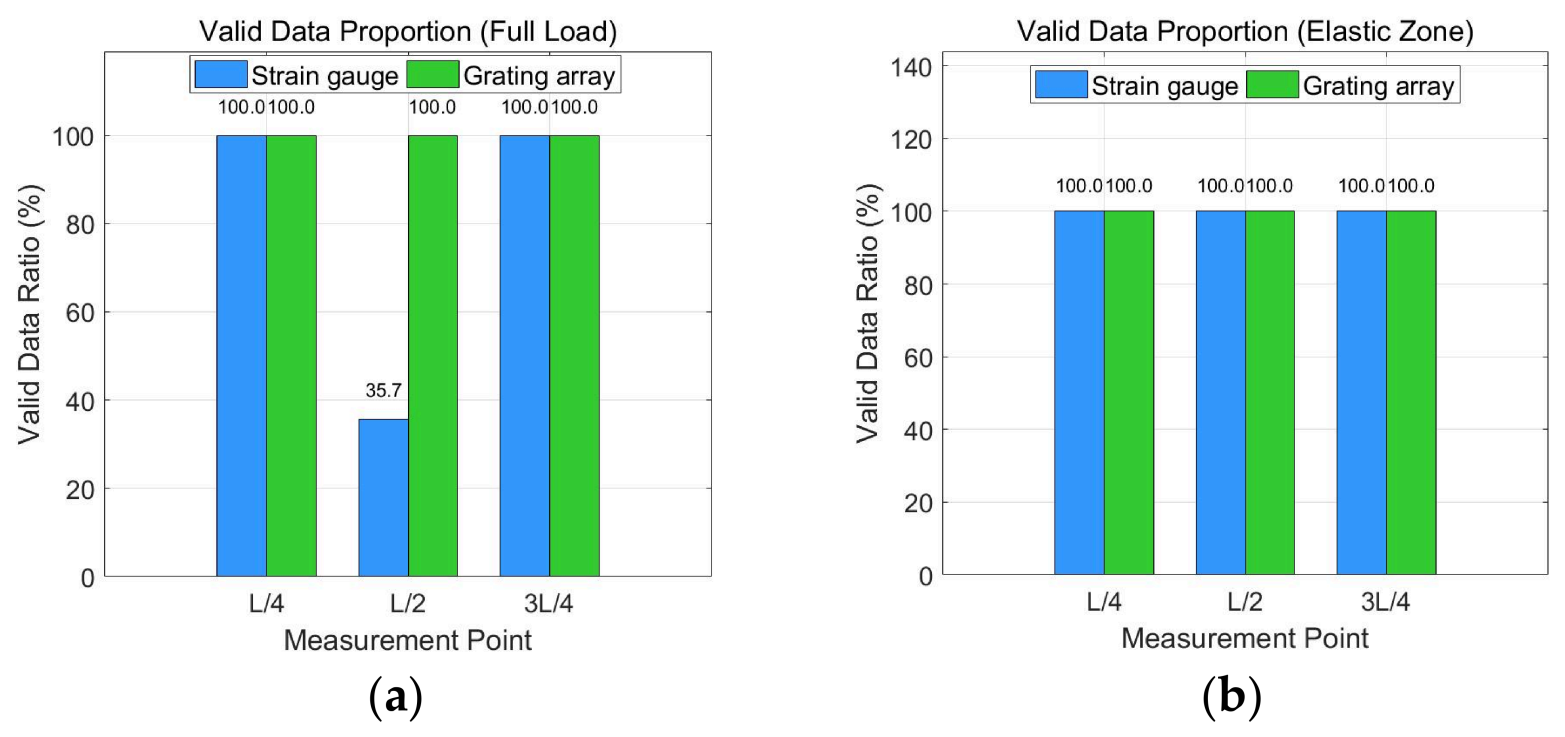
Comparison of valid data proportion between strain gauge and grating array under different loading stages: (**a**) full loading condition (0~1.8P1); (**b**) elastic zone condition (0.3~0.9P1).

However, the coefficient of determination R^2^ alone, though indicative of global linearity, cannot fully reveal local accuracy degradation or extreme outlier risks. To complement this assessment, four supplementary evaluation indicators including Mean Absolute Error (MAE), root mean square error (RMSE), Mean Absolute Percentage Error (MAPE), and the proportion of valid data were calculated for both sensors under two loading ranges: the full loading stage (0–1.8 P1) and the elastic zone (0.3 P1–0.9 P1), with all calculation results visualized in Figure 22, Figure 23, Figure 24 and Figure 25. Focusing on the elastic range (0.3 P_1_–0.9 P_1_), the elastic-stage MAE, RMSE and MAPE values are separately displayed in Figure 22b, Figure 23b and Figure 24b. Within this elastic interval, the UWFBG array achieves lower error indicators with MAE ranging from 63.6 to 79.4 με, RMSE from 65.0 to 79.9 με and MAPE from 19.6% to 32.1%, while the resistance strain gauges deliver higher errors (MAE: 77.4–91.8 με; RMSE: 78.3–102.2 με; MAPE: 21.7–39.7%). Moreover, the valid-data proportion of both sensors stays at 100% in the elastic region (Figure 25b). These results confirm that the UWFBG array provides higher precision and reliability for strain measurement during normal service loads.

#### 4.3.2. Post-Cracking Survivability and Data Continuity (≥1.0 P1)

For loads at and beyond 1.0 P1, the concrete cracks and eventually yields, rendering the elastic theoretical formula invalid. Therefore, the theoretical strain values listed in Table 7 and Table 8 for ≥1.0 P1 are extrapolations shown only to illustrate the onset and progression of nonlinearity; they are not used for quantitative error evaluation. In this regime, the assessment focuses on sensor survivability (the ability to continue delivering valid strain readings) and data continuity.

The full-range (0–1.8 P1) error metrics and valid-data proportions are plotted in Figure 22a, Figure 23a, Figure 24a and Figure 25a. It must be stressed that these “full-range error metrics” combine sensor response with structural nonlinearity and therefore do not represent sensor accuracy in the traditional sense. They are provided to visualize the degradation of strain-gauge signals and the robust continuity of the UWFBG array.

As seen in Figure 25a, the resistance strain gauges at the mid-span sections (both bottom plate and web) experience progressive data loss starting from 1.0P1, with the valid-data proportion ultimately dropping to approximately 35.7 %. In contrast, the UWFBG array maintains a 100% valid-data rate throughout the entire loading sequence, even up to 1.9P1 where the maximum measured strain reaches approximately 8000 με. This demonstrates the exceptional survivability and wide measurement range of the grating array under severe structural damage.

The full-range MAE, RMSE, and MAPE values for the grating array increase substantially at the mid-span under high loads (e.g., MAE up to 1106.4 με, MAPE up to 98.9 %). These large deviations from the elastic extrapolation are a direct consequence of the structural nonlinearity (concrete cracking, reinforcement yielding) that is faithfully recorded by the sensor. They do not indicate a degradation of the sensor’s intrinsic measurement accuracy; rather, they reflect the actual nonlinear strain evolution in the damaged beam. In the same loading stages, the resistance strain gauges fail to deliver any data, depriving engineers of critical information on the structural condition.

Regarding the missing strain gauge data at high loading levels, the primary causes are identified as follows:

1. Strain gauges exceeded their nominal measurement range and entered the plastic stage, leading to irreversible damage to the sensing grid;
2. Local debonding between the gauge and the concrete surface occurred due to large tensile cracking at the mid-span, which disrupted strain transfer;
3. Possible micro-cracking in the vicinity of the gauges introduced additional local strain concentration, causing erratic readings or complete signal loss.

These failure modes are typical for conventional point-type strain gauges under severe overloading conditions, and they explain the observed data gaps.

In the plastic stage, the resistance strain gauges gradually fail with data loss, while the grating array sensors can collect data stably throughout the process without interruption. The maximum measured strain in the test is 8002 με, and the measurement range is not less than 8000 με, showing better continuity and range adaptability. However, the extreme error inflation at the mid-span under full loading also highlights the insufficient measurement accuracy of the grating array in the plastic regime.

Consequently, while the grating array excels in sensitivity and low-noise measurement during elastic deformation, its accuracy degrades significantly under large deformations at the maximum bending moment section; meanwhile, the resistance strain gauges, though reasonably accurate in the elastic zone, are prone to failure under overload conditions. A hybrid strategy—employing grating arrays for high-precision elastic monitoring and resistance strain gauges as supplementary references, together with other redundancy measures—is recommended for future long-term structural health monitoring.

In summary, within the elastic loading regime, the grating array sensing cable exhibits better measurement linearity and closer agreement with theoretical elastic values than resistance strain gauges. In the plastic and failure stages, while the linear relationship between strain and load no longer holds for either sensor, the grating array uniquely maintains continuous data acquisition, demonstrating clear advantages in measurement range and signal survival under large deformations. With the additional error metrics and validity analysis, it is further confirmed that the grating array can accurately characterize the strain response of the π-shaped beam structure within the elastic range, and it still offers data continuity advantages in the plastic stage, albeit with reduced accuracy. The conventional strain gauges, on the other hand, carry the risk of data loss under overload. The combined use of both technologies provides a complementary solution for high-precision, wide-range and high-stability structural strain monitoring.

### 4.4. Discussion on Method Characteristics and Limitations

#### 4.4.1. Spatial Resolution and Crack Identification

The grating array used in this study consists of sensing units with a 1 m gauge length, each of which measures the average strain over that segment. When a single crack occurs within a sensing unit, the resulting stress concentration produces a distinct strain peak in the measured strain profile. This peak, together with comparisons with adjacent units, allows for precise crack localization. Crack width estimation is based on the linear calibration relationship between the FBG peak strain and manually measured crack width established in Section 4.2.4. If a crack happens to lie exactly at the boundary between two adjacent units, both units will record elevated strains, and interpolation can still provide a reasonable location estimate. A more challenging situation arises when multiple fine cracks appear within the same 1 m gauge length. In this case, the sensor delivers only an integrated strain value, and individual crack widths cannot be resolved independently. In our test, the crack spacing during the elastic and early crack-propagation stages was generally larger than 1 m, so the majority of cracks were successfully identified by distinct sensing units. For scenarios with very dense cracking, improving the spatial resolution by reducing the gauge length or combining the array with point-type FBGs at critical locations would be beneficial. Additionally, advanced signal processing techniques, such as deconvolution or machine learning-based pattern recognition, may help to separate the contributions of multiple cracks from the integrated strain signal, representing a direction for future research.

#### 4.4.2. Effect of Debonding on Strain Transfer

The reliability of the FBG array heavily depends on the bond between the optical cable and the concrete surface. If debonding occurs at the fixing points, the strain experienced by the grating will be underestimated or completely lost, potentially leading to undetected cracks. In the present experiment, a high-strength two-component epoxy adhesive was used, and the concrete surface was rigorously cleaned and roughened prior to cable installation. The strain signals remained continuous and stable throughout the entire loading–unloading process, and no abrupt drops or signal anomalies indicative of debonding were observed. For long-term field monitoring, however, environmental factors such as moisture, temperature cycles, and creep may gradually degrade the bond. We therefore recommend the use of protective encapsulation over the bonded area, along with periodic signal integrity checks. Embedding the sensing cable in a shallow groove and covering it with polymer mortar would provide additional mechanical protection and extend the service life.

#### 4.4.3. Comparison with Digital Image Correlation (DIC) and Other Computer Vision Methods

Computer vision techniques, in particular DIC, have become popular for laboratory crack monitoring because they can provide full-field displacement and strain maps with sub-millimeter spatial resolution and enable direct visualization of crack patterns. However, DIC typically requires a stationary camera setup, controlled lighting conditions, and a sprayed speckle pattern on the surface, which limits its applicability to short-term laboratory tests or periodic inspections. In contrast, the UWFBG array can be permanently bonded to the structure and is capable of real-time, continuous monitoring over long periods. It is immune to electromagnetic interference and does not rely on ambient lighting, making it suitable for harsh field environments. Moreover, UWFBG arrays provide absolute, quantitative strain measurements that can be directly compared with theoretical models, whereas DIC requires careful calibration and post-processing to convert pixel displacements into strains. The main limitation of the UWFBG array is its lower spatial resolution (gauge-length dependent), which can be partly mitigated by increasing the grating density. The two methods are highly complementary: DIC can be used during laboratory experiments to capture fine details of crack initiation and propagation for validation purposes, while the UWFBG array can be deployed for long-term structural health monitoring in the field. Integrating both techniques into a hybrid monitoring framework would combine the strengths of high-resolution surface visualization and robust, long-term strain measurement, offering a more comprehensive assessment of structural integrity.

#### 4.4.4. Limitations of the Experimental Campaign

It should be expressly noted that the experimental results presented in this study derive from a single full-scale prestressed π-beam subjected to a single monotonic step-loading protocol. While the test beam is representative of the prefabricated girders used in the Changshengkou Bridge project and the loading sequence follows standard code-based procedures, the generalisability of the quantitative crack–width correlation (ε = 7918·w − 110, R^2^ = 0.971) and the observed sensor performance characteristics remains constrained. Factors such as variations in concrete material properties, prestressing levels, environmental conditions, and loading history can influence crack evolution and strain transfer. Consequently, the present findings should be regarded as a proof-of-concept demonstration; repeated experiments on multiple beams and under diverse loading scenarios are desirable to establish statistically robust performance benchmarks and to translate the methodology into routine field practice.

## 5. Conclusions

This study presents an experimental investigation using a UWFBG array for online monitoring of crack evolution in a full-scale prestressed π-beam. The main findings are:

1. Distributed strain monitoring: The UWFBG array successfully captured the entire crack evolution process—from initiation at 0.9P_1_ to complete failure at 1.9P_1_—with stable signal acquisition throughout.
2. Crack identification and tracking: The transformation of strain–load curves from linear-to-nonlinear and the triangular-shaped spatial strain distribution clearly indicated crack initiation, propagation, and failure, matching manual observations.
3. Quantitative performance evaluation: Compared to resistance strain gauges, the UWFBG array delivered higher linearity in the elastic stage (R^2^ > 0.99) and, more importantly, provided uninterrupted data collection over the full loading range without any sensor failure. While its strain–load linearity degraded after concrete cracking due to structural nonlinearity, the continuous strain record enabled reliable tracking of damage progression, which was not possible with the strain gauges that progressively lost signal. More importantly, quantitative correlation analysis revealed a strong linear relationship between UWFBG peak strains and manually measured crack widths (ε = 7918·w − 110, R^2^ = 0.971), demonstrating that the system can provide not only spatial crack location but also a reliable estimate of crack severity, bridging the gap between distributed sensing and engineering condition assessment, though its direct transfer to other structures is not advisable without independent calibration.
4. Practical potential: The system shows great promise for long-term bridge health monitoring, though spatial resolution and bond durability need further improvement for dense cracking scenarios. The established strain–crack width calibration curve provides a practical basis for estimating crack severity directly from distributed strain data in future field applications.

It is acknowledged that the experiments were performed on a single test specimen; further validation on additional beams and under varied loading conditions is needed to confirm the broader applicability of the presented results and the proposed empirical strain–crack–width relationship.

## Figures and Tables

**Table 1:** Characteristics of the π-shaped beam during the full load-bearing stage.

Loading Stage	Load Range	Core stress Characteristics	The Law of Strain Variation
Elastic stage	P < Pcr	The concrete did not crack. The stress distribution of the cross-section conformed to the assumption of a flat section. The tensile zone concrete stress did not reach the tensile strength. The overall stiffness of the beam remained constant.	The strain increases in a strictly linear manner with the load, maintaining a constant slope and without any sudden changes.
Crack initiation stage	P = Pcr	The tensile strength of the concrete in the tensile zone reached the limit. The first stress crack emerged. The stress concentration occurred at the crack tip, and the stress distribution of the cross-section redistributed.	The strain growth rate suddenly accelerates, and a nonlinear inflection point appears in the curve. The strain value deviates from the theoretical linear value.
Crack propagation stage	Pcr < P < Pu	The number, length, and width of the cracks continued to increase. The cracks transformed from L-shaped to U-shaped and penetrated through. The area of the compressive zone gradually decreased, and the stiffness of the beam gradually decreased.	The strain increases nonlinearly with the load, and the strain growth rate is much greater than the load growth rate.
Plastic deformation stage	P ≈ Pu	The beam entered a plastic stress state. The concrete in the compressive zone approached the limit compressive strain. The reinforcing bars in the tensile zone began to yield, and the bending moment remained basically constant.	The strain continues to increase significantly, while the load growth is slow. The slope of the strain–load curve tends to stabilize but remains nonlinear.
Complete failure stage	P > Pu	The concrete in the stressed area has fractured and the cracks have completely penetrated. The beam loses its bending-bearing capacity, and the structure is completely destroyed.	There is no upper limit for the strain increase, and after the load reaches its peak, it rapidly decreases.

Note: (1) Pcr represents the critical load for crack initiation. (2) Pu represents the ultimate load.

**Table 2:** Load Application Grading in the Test (Unit: kN).

Load Classification	Load Factor	Load Value
Level 0	0.3P1	283
Level 1	0.5P1	471.5
Level 2	0.7P1	660.1
Level 3	0.8P1	754.4
Level 4	0.9P1	848.7
Level 5	1.0P1	943.0
Level 6	1.1P1	1037.3
Level 7	1.2P1	1131.6
Level 8	1.3P1	1225.9
Level 9	1.4P1	1320.2
Level 10	1.5P1	1414.5
Unload	0	0
Level 11	1.6P1	1508.8
Level 12	1.7P1	1603.1
Level 13	1.8P1	1697.4
Level 14	1.9P1	1791.7

Note: (1) P1 represents the maximum test load value corresponding to the design resistance value of the structure under the ultimate limit state of bearing capacity (943.0 kN).

**Table 3:** Configuration of UWFBG Array Strain Sensing Cables.

Strain Sensing Cable Serial Number	Grating ID Range	Physical Location	Monitored Structural Part
1	11–17–23	Left web, inner side	Web plate, left side
2	38–32–26	Left web, outer side	Web plate, left side
3	42–47–53	Bottom plate, left–inner	Bottom slab, left of center
4	68–62–56	Bottom plate, left–outer	Bottom slab, left of center
1′	72–77–83	Right web, inner side	Web plate, right side
2′	98–92–86	Right web, outer side	Web plate, right side
3′	101–107–113	Bottom plate, right–inner	Bottom slab, right of center
4′	130–123–117	Bottom plate, right–outer	Bottom slab, right of center

Note: (1) Grating IDs are given as Head–Middle–Tail. The approximate mid-span crack monitoring points correspond to the middle IDs.

**Table 4:** Main technical parameters of the UWFBG array sensing system.

Parameter	Value
Demodulator Model	FL-S-3000
Number of channels	4
Sampling Frequency	10 Hz
Wavelength Resolution	1.0 pm
UWFBG Array Strain Sensing Optical Cable	FL-DSC-0100-SM
Grating Reflectivity	≈0.01%
Grating Spacing	1.0 m
Grating Gauge Length	1.0 m
Spatial Resolution	1.0 m
Measurement Range	±8000 με
Central Wavelength	≈1550 nm
Typical Signal-to-Noise Ratio	>20 dB
Nominal Strain Resolution	≈0.83 με
Manufacturer (Demodulator/Cable)	FENGLI Optoelectronics Technology Co., Ltd., Wuhan, China

**Table 5:** Left crack of 848.7 kN (0.9P1).

Crack Number	Type of Change New Addition	Type of Cracks	Total Length of the Crack (cm)	Crack Width (mm)	Crack Description
1	New addition	L-shaped cracks	32	0.04	At the middle section of the span, 0.50 m from the side of the smaller pile number, L outer flange = 12 cm, L floor plate = 20 cm, W = 0.04 mm
2	New addition	L-shaped cracks	40	0.09	At the middle section of the span, 0.30 m from the side of the smaller pile number, L outer flange = 20 cm, L floor plate = 20 cm, W = 0.09 mm
3	New addition	Vertical cracks	4	0.06	At the large pile number side of the mid-span, 0.20 m,
4	New addition	L-shaped cracks	39	0.06	L outer flange = 4 cm, W = 0.06 mm
5	New addition	L-shaped cracks	20	0.06	At the mid-span, 0 m, L inner flange = 22 cm,
6	New addition	L-shaped cracks	20	0.06	L floor plate = 17 cm, W = 0.06 mm
7	Type of Change: New addition	L-shaped cracks	28	0.06	At the large pile number side of the mid-span, 0.50 m, L inner flange = 15 cm, L floor plate = 5 cm, W = 0.06 mm

**Table 6:** Summary of crack characteristics at different loading stages.

Loading Stage	Load Level	Number of Cracks	Maximum Crack Width (mm)	Maximum Crack Length (cm)	Dominant Crack Type
Initiation	848.7 kN (0.9P1)	11	0.11	40	L-shaped
Expansion	1.0P1–1.9P1	Increased from 11 to 90	Increased from 0.11 to 1.21	Increased from 40 to 246.5	Evolved from L-shaped to U-shaped and penetrating
Ultimate	1791.7 kN (1.9P1)	90	1.21	246.5	U-shaped and penetrating (web and bottom plate)

**Table 7:** Comparison of test results between UWFBG Array and Strain Gauges (bottom plate).

Loading Level	L/4 Measurement Point			L/2 Measurement Point			3L/4 Measurement Point		
	Theoretical Value	Strain Gauge	UWFBG Array	Theoretical Value	Strain Gauges	UWFBG Array	Theoretical Value	Strain Gauges	UWFBG Array
0.3P1	112	56	52	224	117	138	112	37	62
0.5P1	187	118	128	374	296	302	187	129	136
0.7P1	261	170	199	523	551	452	261	186	202
0.8P1	299	183	221	598	645	505	299	206	224
0.9P1	336	209	250	672	850	597	336	250	253
1.0P1	374	228	293	747	---	773	374	280	293
1.1P1	411	227	322	822	---	1039	411	290	320
1.2P1	448	280	378	896	---	1740	448	296	395
1.3P1	486	291	404	971	---	2040	463	297	421
1.4P1	523	317	431	1046	---	2372	499	296	449
1.5P1	560	373	474	1121	---	3034	535	303	499
1.6P1	598	406	501	1195	---	3510	598	318	501
1.7P1	635	462	536	1270	---	4138	635	364	551
1.8P1	672	544	589	1345	---	5860	672	672	544

Note: (1) For loads ≥ 1.0 P1, the theoretical strain values are extrapolated from the elastic formula and are provided only to illustrate the deviation caused by cracking; they do not represent the real structural strain and are not used for quantitative error evaluation. All sensor accuracy metrics (R^2^, MAE, RMSE, MAPE) reported in this section are calculated solely from the 0.3 P1–0.9 P1 elastic data.

**Table 8:** Comparison of test results between UWFBG Array and Strain Gauges (web plate).

Loading Level	L/4 Measurement Point			L/2 Measurement Point			3L/4 Measurement Point		
	Theoretical Value	Strain Gauge	UWFBG Array	Theoretical Value	Strain Gauges	UWFBG Array	Theoretical Value	Strain Gauges	UWFBG Array
0.3P1	99	42	33	198	93	124	99	37	65
0.5P1	165	101	93	331	223	263	165	117	143
0.7P1	232	152	147	463	338	394	232	170	206
0.8P1	265	160	162	529	318	442	265	184	229
0.9P1	298	179	185	595	301	526	298	212	260
1.0P1	331	194	217	662	297	697	331	229	301
1.1P1	364	191	241	728	---	922	364	250	331
1.2P1	397	212	290	794	---	1611	391	295	341
1.3P1	430	215	310	860	---	1892	424	294	366
1.4P1	463	217	325	926	---	2241	456	306	386
1.5P1	496	228	349	992	---	2905	489	332	443
1.6P1	529	230	372	1059	---	3352	529	373	562
1.7P1	562	222	399	1125	---	3974	562	483	637
1.8P1	595	202	425	1191	---	5471	595	595	202

Note: (1) For loads ≥ 1.0 P1, the theoretical strain values are extrapolated from the elastic formula and are provided only to illustrate the deviation caused by cracking; they do not represent the real structural strain and are not used for quantitative error evaluation. All sensor accuracy metrics (R^2^, MAE, RMSE, MAPE) reported in this section are calculated solely from the 0.3 P1–0.9 P1 elastic data.

**Table 9:** Linearity (R^2^) of strain–load fitting in the elastic stage (0.3 P1–0.9 P1) for resistance strain gauges and UWFBG array at key sections.

Sensor	Bottom Plate Linearity (R^2^)			Web Plate Linearity (R^2^)		
	L/4	L/2	3L/4	L/4	L/2	3L/4
Strain Gauge	0.9891	0.9831	0.9844	0.9769	0.8029	0.9782
FBG Array	0.9932	0.9972	0.9940	0.9910	0.9975	0.9934

## Data Availability

The data presented in this study are available on request from the corresponding author.

## Abbreviations

The following abbreviations are used in this manuscript:

FBG	Fiber Bragg Grating
UWFBG	Ultra-weak Fiber Bragg Grating
EC	Elastic modulus of concrete
Pcr	Critical load for crack initiation
Pu	Ultimate load
P1	Maximum test load corresponding to design resistance
JTG 3362-2018	Design Specifications for Highway Reinforced Concrete and Prestressed Concrete Bridges and Culverts
UPV	Ultrasonic testing. Ultrasonic pulse velocity
MAE	Mean Absolute Error
RMSE	Root Mean Square Error
MAPE	Mean Absolute Percentage Error
DIC	Digital Image Correlation
WDM	wavelength-division multiplexing
DOFS	distributed optical fiber sensing
OFDR	optical frequency domain reflectometry
TDM	time-division multiplexing
